# Nanomedicine in Neuroprotection, Neuroregeneration, and Blood–Brain Barrier Modulation: A Narrative Review

**DOI:** 10.3390/medicina60091384

**Published:** 2024-08-24

**Authors:** Antea Krsek, Ana Jagodic, Lara Baticic

**Affiliations:** 1 Faculty of Medicine, University of Rijeka, 51000 Rijeka, Croatia; antea.krsek@uniri.hr; 2 Department of Family Medicine, Community Health Center Krapina, 49000 Krapina, Croatia; ana.jagodic@dzkzz.hr; 3 Department of Medical Chemistry, Biochemistry and Clinical Chemistry, Faculty of Medicine, University of Rijeka, 51000 Rijeka, Croatia

**Keywords:** BBB (blood–brain barrier), nanofiber scaffolds, nanomedicine, nanomaterials, nanoparticles, neuronal regeneration

## Abstract

Nanomedicine is a newer, promising approach to promote neuroprotection, neuroregeneration, and modulation of the blood–brain barrier. This review includes the integration of various nanomaterials in neurological disorders. In addition, gelatin-based hydrogels, which have huge potential due to biocompatibility, maintenance of porosity, and enhanced neural process outgrowth, are reviewed. Chemical modification of these hydrogels, especially with guanidine moieties, has shown improved neuron viability and underscores tailored biomaterial design in neural applications. This review further discusses strategies to modulate the blood–brain barrier—a factor critically associated with the effective delivery of drugs to the central nervous system. These advances bring supportive solutions to the solving of neurological conditions and innovative therapies for their treatment. Nanomedicine, as applied to neuroscience, presents a significant leap forward in new therapeutic strategies that might help raise the treatment and management of neurological disorders to much better levels. Our aim was to summarize the current state-of-knowledge in this field.

## 1. Introduction

Neurodegenerative diseases and traumatic brain injuries represent one of the major challenges for healthcare systems worldwide. These disorders usually progress to neuron loss/damage and further lead to debilitating cognitive and motor deficiencies. Formal therapeutic strategies have, so far, failed to elicit neural regeneration, and thus functionality, due to the intrinsic intricacy of the nervous system itself and the protective but restrictive nature of the blood–brain barrier (BBB) [1]. Given the increasing difficulty of developing effective neurorehabilitation strategies, there is a growing need for generating personalized medicine. Steps toward the future of medicine have led to the development of cutting-edge techniques, such as nanoparticles as part of nanomedicine and bioengineering.

Nanotechnology is a fast-evolving field for trials in neural restorative therapies. Due to the distinctive properties of nanomaterials and their ability to interact at cellular and molecular levels, they can provide new opportunities for the growth and repair of neural tissue. Such materials could be fine-tuned to provide structural support, deliver therapeutic agents, or induce calculated enhancements of indigenous restorative mechanisms within the central nervous system (CNS) [2]. 

Combining biological components with materials used in tissue healing has given rise to tissue engineering, a promising effective approach. Creating methods to stop fibrosis and inflammation, while introducing foreign materials that can serve as scaffolds for cell development, is the main goal of brain tissue engineering. To provide a biomimetic environment that supports neuronal growth, these scaffolds must mimic the extracellular matrix seen in nature. Furthermore, throughout the implantation procedure, the scaffolds need to retain their stability and structural integrity [3,4]. 

The optimal scaffold for neural tissue engineering should possess several key characteristics: it must be non-toxic, promote cell survival, proliferation, and migration, facilitate electrochemical communication between cells, mimic the mechanical properties of brain tissue, and ideally, allow for the controlled release of substances [5].

The blood–brain barrier forms a very important part of protecting the brain, modulating the entry of substances, and, in the process, maintaining an important stable environment crucial for neural function. The BBB develops between the endothelial cells, as tight junctions, and through specialized transport systems to block the infiltration of damaging toxins and pathogens inside the brain. Indeed, selective permeability poses a major challenge in therapeutic agents’ distribution for the treatment of neurological disorders as well as neural regeneration [6,7,8]. The BBB is an integral part of the defense against poisonous substances and anchors the stable environment in which neural function can take place. At the same time, though, this very selective permeability creates a formidable barrier to the distribution of therapeutic agents working toward neural regeneration. Strategies that transiently safely open the BBB could permit the targeted delivery of regenerative compounds without causing damage to the protective barrier [9,10]. 

Recent developments in nanomedicine are starting to present innovative ways of overcoming this challenge. A range of techniques that might transitively safely open the BBB, allowing targeted delivery of regenerative therapies, while restoring normal BBB functions and integrity, should be considered. Among these modalities, focused ultrasound is performing this remarkable feat when used with microbubbles, at present. It allows effective drug and nanoparticle delivery so that the brain is reached without permanent injury [11]. 

Another approach includes the use of chemo-modulators, inducing controlled permeability of the BBB. Molecules, such as bradykinin or hypertonic solutions, cause reversible changes in tight junctions, making it possible to pass the therapeutic agents without significant risks [12]. Nanoparticles designed for receptor-mediated transcytosis or guided by a magnetic field second to none nanotechnology enhance modulation of the BBB. The nanoparticles can be programmed to carry or target therapeutic drugs or genetic material to specific brain areas or regions of interest [13]. 

Neuroprotection is indispensable for the reduction of progressive damage in neurodegenerative diseases and during events after brain injuries. Oxidative stress and inflammation are major contributors to neuronal damage and cell death [14,15]. Neuroprotective nanomaterials can, therefore, offer targeted delivery of antioxidants and anti-inflammatory agents, attacking these fatal pathological processes at their very roots.

Nanoparticles could be designed to penetrate the BBB and deliver therapeutic agents precisely to the affected areas of the brain, providing localized, long-term release. This model strategy not only increases the efficacy of the treatment but also brings down the systemic side effects. Nanotechnology has the potential for not only protecting existing neurons through neuroprotective strategies but also combining these with regenerative therapies—ultimately repairing and regenerating neural tissue that has been damaged.

Here, the area of neuroprotection research investigating how to use nanoparticles for delivering various drugs and agents that could help attenuate neuronal damage is also considered. Case studies and examples from real life are presented about the potential impact these advanced approaches in nanotechnology have. Finally, future directions for the crossroads of BBB modulation and neural regeneration/neuroprotection treatments are considered. The latest studies on such strategies and nanotechnology advances are addressed as well, highlighting how such innovations can eventually make treatments against neurodegenerative disorders and brain trauma more effective and far-reaching. Using the unique capabilities of nanomaterials, we can get closer to breakthroughs in neural regeneration and neuroprotection.

The present review considers the multifaceted role of nanotechnology in neural regeneration and neuroprotection. The role of nanofiber scaffolds and nanoparticles in the differentiation of neural stem cells is discussed. We elaborate on the challenges raised by the BBB to the conveyance of therapeutics to the brain, including fresh strategies implicated in BBB modulation using nanoparticles. We further illustrate advantageous effects resulting from the combination of BBB modulation with therapies of neural regeneration.

## 2. Nanotechnology: Innovative Synthesis Techniques and Health Applications

**Figure 1:**
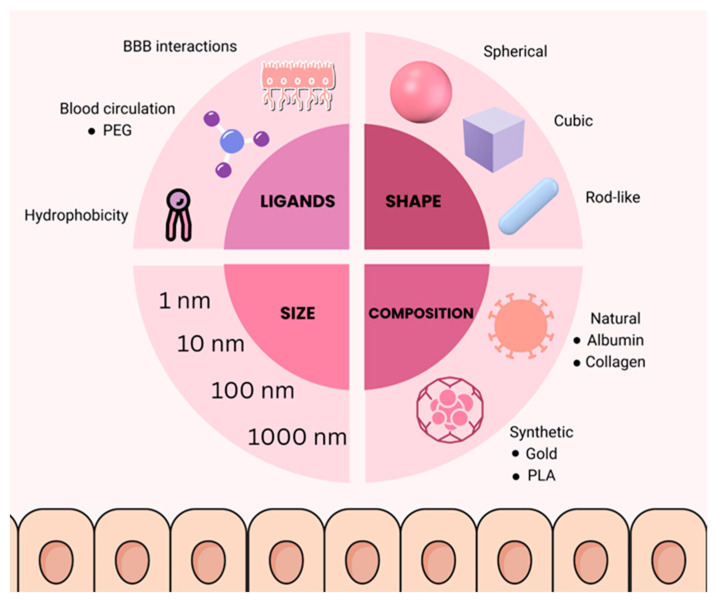
Nanoparticle characteristics affecting drug-delivery outcomes.

Nanoscience and nanotechnology, the study of structures and systems between 1 and 100 nm, opened opportunities for many technologies to follow and brought changes into many different fields of study. Among them are those concerning health, such as cancer treatment. Popular awareness and commercial impact date back to the beginning of 2000 and are, hence, quite exemplary of the development and principles in these fields [16]. Essential design parameters for nanoparticle-based drug delivery, in this way, will be such parameters as size, form, composition, and, finally, surface features, and the latter demands surface functionalization, which is of key importance for targeted delivery [17]. In effective neuron-targeting drug delivery, the design of nanomedicines plays a critical role, particularly regarding the selection of ligands, as well as the size and shape of the nanoparticles. These parameters are tailored to optimize the targeting, internalization, and therapeutic impact of the drug within the CNS. Peptide ligands, antibodies, aptamers, and small molecules offer various advantages depending on the target receptor and desired specificity. In terms of size, nanoparticles within the range of 10 to 100 nm are generally most effective for BBB penetration and neuronal uptake. However, particles at the lower end of this spectrum (e.g., 10–20 nm) are often more successful in penetrating the BBB due to their ability to exploit transcytosis mechanisms without being cleared by renal filtration. Larger nanoparticles, within the range of 50 to 100 nm, often have longer circulation times due to reduced renal clearance. This increases the likelihood of reaching the CNS, though it may also increase off-target accumulation. The shape of nanoparticles influences their cellular uptake, circulation dynamics, and ability to cross biological barriers. Shape optimization, whether spherical, rod-shaped, cubic, or disc-like, further influences how these nanoparticles behave in the body, affecting their ability to deliver therapeutic agents directly to neurons [16,17]. Nanoparticle characteristics affecting drug-delivery outcomes are presented in Figure 1.

Many of the new devices and patentable technologies are based on nanomaterials whose applications are generally controlled by the synthesis and growth mechanisms of the nanostructure. A nanomaterial suitable in one application may be more effective in another if synthesized differently. Nanomaterials’ structure and morphology are also dependent on the way they are synthesized [18]. We can differentiate two types of nanotechnology: bottom-up and top-down. The techniques of bottom-up and top-down approaches vary in their methods of fabrication. Bottom-up techniques create nanostructures from atomic or molecular building blocks, thereby allowing very fine control of the structure and composition. On the other hand, top-down techniques start with larger materials and reduce them to nanoscale dimensions via techniques inherently associated with limited atomic arrangement control [19]. Some of the methods in top-down approaches are mechanical milling, laser ablation, and electrospinning for nanofiber production. One of the inexpensive methods to produce nanoscale materials by grinding bulk substances is mechanical milling. It turned out to be effective for the preparation of blends of different phases and nanocomposites. Among these key products of the process are advanced materials, including oxide- and carbide-strengthened aluminum alloys, nanoalloys, and carbon nanomaterials, which could potentially be applicable in environmental cleanup, energy storage, and its conversion [20,21]. The high energy of the laser vaporizes the material to form nanoparticles in a process called laser ablation, bypassing stabilizers. This method yields diverse nanomaterials in an environmentally friendly way and provides control over the colloidal solution particle properties by tuning the laser parameters [22]. 

One of the most popular methods to produce polymer nanofibers with huge biomedical application potential is electrospinning. Nanofibers have nanometer-scale diameters that provide larger surface areas than microfibers, and thus their surface can be functionalized. Electrospinning can convert a variety of polymers into nanofibers, and such technology has been used in several applications related to tissue engineering, drug delivery, and sensors [23]. Electrospinning is utilized for generating nanofibers through electrostatic forces that stretch and solidify a polymer solution. During the process, a syringe pump controls the circulation of the polymer solution through a conductive needle, and a high-voltage power supply induces an electric field in between the needle and a collector. This will induce charges on the solution and formation of the Taylor cone, which will consequently result in polymer jet ejection. While the jet advances to the collector, the solvent dissipates, causing the polymer to harden into nanofibers. Although conceptually simple, the process of electrospinning often becomes quite demanding in setup and adjustment, with different polymers for the optimization of fiber properties and applications [24,25].

### 2.1. Bottom-Up Approaches

Some of the methods in bottom-up approaches are the sol-gel processes, chemical vapor deposition (CVD), and atomic layer deposition (ALD). The sol-gel process is the cost-effective chemical route for synthesizing metal oxide nanoparticles. The techniques involve the dissolution of a precursor in water or alcohol and then hydrolysis or alcoholysis, which forms a gel, followed by drying and calcination of the gel. The method offers very good control over the chemical composition. Applications include ceramics and thin films, and industries utilizing this are those involved in optics, electronics, and pharmaceuticals. This may affect the properties of the gel, and the dried gel may be ground into nanoparticles for specialized applications [26,27]. 

Chemical vapor deposition of thin films and coatings is one important technology delivering high-quality products. It is developing toward the use of new materials, particularly 2D materials, for instance, graphene and transition metal dichalcogenides (TMD), and high-purity polymeric films. Herein, the authors describe the fundamentals of techniques in CVD, including equipment, regulation of processes, material analysis, and consistency. We discuss effective methods in substrate preparation, high-temperature synthesis, and post-processing techniques, illustrated through the cases of graphene and TMDs. Recent achievements and challenges toward scale-up are presented, emphasizing upcoming advancements in plasma reactor design aimed at enhancing high-throughput and low-temperature thin-film production [28,29]. ALD is a technique in which ultra-thin films deposit with perfect control of the thickness, even on complicated 3D surfaces, promoting efficiency in electronic devices. Advanced nanopatterning has largely employed ALD in microelectronics, energy storage, desalination, catalysis, and several other fields. Atomic layer deposition is a technique using chemical reactions between two or more precursors that are alternately introduced into a chamber with the substrate at a certain temperature and pressure, and in this way, it deposits the material layer by layer. In contrast, chemical vapor deposition pumps the precursors at the same time, whereas ALD pulsates the precursors, hence its name [30,31].

### 2.2. Green Synthesis Methods

The environmentally friendly production of nanoparticles using plant extracts, fruit juices, and other natural sources is environmentally sustainable, budget-friendly, and simple to manufacture [32]. The nanoparticles obtained from these extracts have exhibited promising antiviral and antimicrobial properties with potential for treating infections [33]. Synthesis through natural materials or biologically active substances is an established and continuous research area aiming toward enhancing production processes to reduce their impact on the environment while meeting growing demands [34]. It is, in most cases, cost-effective and faster due to the possibility of carrying out a one-step process while maintaining or enhancing the properties of nanoparticles. It makes it possible to produce nanoparticles of various dimensions and forms, thereby remaining very attractive and viable for synthesizing nanostructures and other compounds in a more sustainable way [35]. Plant extracts of leaves, roots, flowers, etc., are used to produce NPs in moderate conditions and are environmentally friendly. Such biogenic NPs characterized by different techniques showed good anticancer and antioxidant activities and were quite stable in different conditions. Asteraceae plants showed their potency to create a range of NPs, such as silver and gold nanoparticles (AuNPs), with different applications. The exact mechanisms of phytochemicals in the synthesis of NPs are still unknown, and this was identified as the focus of the research in phyto-nanotechnology [36].

### 2.3. Electrospun Nanofibrous Frameworks for Nerve Tissue Regeneration

Prevailing methods for nerve repair, such as autografts and allografts, face significant limitations. Autografts, while effective, are constrained due to complications at the donor site and restricted availability [37]. Allografts, on the other hand, suffer from issues related to immune rejection and inadequate functional integration [38]. Additionally, traditional approaches often fail to address the complexity of nerve regeneration, such as guiding axon growth and providing a supportive microenvironment [39].

Electrospun scaffolds offer a novel solution by overcoming these limitations. Unlike conventional methods, electrospun scaffolds can be precisely designed to replicate the extracellular matrix (ECM), providing an ideal environment for cell attachment and growth [40]. Their high surface area and tunable porosity facilitate better cell infiltration and axon guidance [41]. Furthermore, electrospun nanofibers can be enhanced with growth factors or conductive materials to enhance neuronal regeneration and improve integration with native tissues [42]. This innovative approach addresses both the structural and biochemical needs of nerve repair, offering a promising alternative to traditional methods.

Macrophages are crucial contributors to the initial reaction to nerve injury and scaffold implantation. They are critical for clearing debris and secreting cytokines that modulate inflammation and guide tissue repair [43]. Electrospun scaffolds can be engineered to support macrophage functions by incorporating bioactive molecules that modulate their inflammatory response and promote a transition from a pro-inflammatory state to a reparative state [42]. This interaction helps generate a more beneficial environment for subsequent cell recruitment and tissue regeneration.

Schwann cells (SC) are vital for peripheral nerve repair, as they support axon regeneration and remyelination [44]. Such modifications to the surface or incorporation of signaling molecules in electrospun scaffolds have demonstrated the ability to enhance SC adhesion, proliferation, and migration [42]. In the case of electrospun scaffolds with aligned nanofibers, specific advantages for SCs are provided by physical cues that guide growth and align axons, thereby restoring nerve function [45]. Whether NSCs or MSCs (mesenchymal stem cells), stem cells have been imperative for long-term nerve regeneration since they can proliferate and transform into different neuronal and glial cell types [46]. 

Overall, the interaction between these cell types and electrospun scaffolds is crucial for optimizing nerve repair and regeneration. By carefully designing scaffolds that support macrophage activation, SC function, and stem cell differentiation, researchers can significantly improve outcomes in nerve tissue engineering.

Numerous physiological processes, including respiration, muscular contraction, and sensory perception, are mostly regulated by the neurological system. Nonetheless, it is still difficult to recover from severe nerve injuries, which include PNIs (peripheral nerve injuries) that occur outside of the CNS and CNS traumas that affect the brain and spinal cord. Neural circuits can be disrupted by degenerative illnesses and CNS traumas, which may lead to impaired neuronal function and, in severe situations, death [47]. About five million new cases of PNI are documented annually, accounting for roughly 3% of all injuries. The condition causes neuropathic pain, defective innervation regions, and abnormalities in motor or sensory function. Although secondary damage and donor nerve shortage are major obstacles to autogenous nerve transplantation, it has historically been utilized extensively for nerve repair [48,49]. Scaffolds created through tissue engineering have been investigated as potential substitutes for directing nerve regeneration.

Numerous techniques, including phase separation, self-assembly, and template synthesis, have been investigated for the production of nanofibers. Due to its benefits over other methods, including exact control over fiber structure and diameter, electrospinning has attracted a lot of interest [50,51]. With a large surface area for cell adhesion, a porous shape to facilitate cell infiltration, and tunable mechanical characteristics for the best possible physiological responses, electrospun micro/nanofibers imitate the ultrastructure of the ECM in nature [49,52].

Using a needle to inject a polymer solution, an electrospinning technique creates a suspended conical droplet that, when subjected to an electric field, shoots jets. On a collection plate, fibers are left behind when the solvent evaporates. For all their benefits, standard electrospun scaffolds are not always effective in mending large-scale nerve lesions because of problems such as irregular cell migration, poor electrical stimulation, insufficient growth factors, and adverse immuno-inflammatory reactions [53].

Numerous strategies have been utilized to improve electrospinning scaffolds for nerve regeneration to overcome these obstacles. For example, aligned electrospun nanofibers have shown promise in directing axon elongation and neurite formation. Growth factors and surface changes have the potential to boost neuron regeneration, decrease immuno-inflammatory reactions, and increase cell proliferation. Furthermore, the development and proliferation of neurons have been stimulated by electrical stimulation [42]. Through immunological regulation and paracrine signaling, interactions between macrophages and stem cells (SCs) have been investigated to support nerve regeneration [54,55]. Antioxidant-based strategies have been established to assist nerve tissue healing by mitigating nerve scarring and neutralizing reactive oxygen species (ROS) [56].

## 3. Important Materials

### 3.1. Biodegradable Polymers

#### 3.1.1. Polycaprolactone

Polycaprolactone (PCL) has been described as a semi-crystalline, hydrophobic, and biodegradable polymer in our earlier studies [57]. Nerve guidance conduits (NGCs) have been built using electrospun PCL nanofibers, which replicate the architecture of the ECM present in peripheral nerves [58,59]. Additionally, these electrospun PCL fibers have been employed to help embryonic stem cells differentiate into neural lineages. Compared to randomly oriented fibers, aligned PCL fibers have been produced in the domain of neural tissue engineering to yield topographical signals that increase axon extension and direct axon development more efficiently [60,61]. However, the limited applicability of pure PCL stems from its sluggish breakdown kinetics and strong hydrophobicity. Moreover, its standalone relevance is further restricted by its reduced cell activity and vulnerability to plasma proteins [62]. Therefore, PCL is frequently co-electrospun with other polymers to speed up polymer degradation, enhance wettability, and improve biocompatibility [63].

#### 3.1.2. Poly(Lactic-Co-Glycolic Acid) (PLGA)

A biodegradable substance called polylactic acid (PLA) is produced from renewable plant sources, including cassava and maize. Tissue engineering scaffolds make considerable use of PLA-based electrospun materials because of their exceptional machinability and flexible mechanical strength [64,65]. Nevertheless, difficulties with cell adhesion, migration, proliferation, and differentiation arise from PLA’s hydrophobic surface, which is abundant in methyl groups [66]. Additionally, when PLA degrades, acidic byproducts are produced that have the potential to cause inflammation [67]. An aliphatic polyester, known as polyethylene glycolic acid (PGA), is hydrophilic and capable of biodegradation, and electrospun PGA fibers break down more quickly than PLA. This characteristic facilitates the migration of SCs and the early Büngner band development [68,69].

The Food and Drug Administration (FDA) has authorized poly(lactic-co-glycolic acid) (PLGA), a copolymer comprising PLA and PGA joined by ester linkages. By changing the PLA-to-PGA ratio, PLGA-based scaffolds’ rate of degradation may be regulated. It can form membranes, has low toxicity, and strong mechanical strength [70,71]. Electrospun scaffolds based on PLGA that have aligned orientations and suitable fiber sizes promote nerve cell adhesion and proliferation, which in turn directs the formation of neurites outward. On the other hand, as with PLA, PLGA generates products of acidic breakdown that might result in aseptic inflammation. Moreover, its hydrophobic characteristics diminish biocompatibility [66].

#### 3.1.3. Conducting Polymers 

A rapidly developing field of study in brain tissue engineering is electrospun scaffolds composed of conducting polymers (CPs) [72]. Highly conjugated σ orbital overlaps throughout the backbone are made possible by the σ bonds in CPs, which are weaker than the σ bonds in the polymer chain and are distinguished by their loosely bound electrons in the backbone [73,74]. A neutral polymer chain can go through oxidation or reduction to enter a highly conductive state, which promotes nerve-derived cell adhesion, proliferation, infiltration, and directional migration [75]. Electronegative nerve cells produce endogenous electric fields, which are amplified by cell proliferation on conductive surfaces [76,77]. To promote neuron regeneration, researchers have investigated electrospinning CPs into nanoscale and microfibers [76,78]. Polypyrrole (PPy) and polyaniline (PANI) are two typical CPs, which are usually dissolved in organic solvents to be electrospun. Based on the polymerization of aniline monomers, PANI is formed in totally oxidized, partially oxidized, and reduced forms. The intermediate oxidation state of PANI is being especially considered for its reliability, electrical conductivity, and redox tunability. The nitrogen atoms of quinone diimine fragments are protonated by doping with protic acids, such as hydrochloric acid, sulfuric acid, or sulfonic acid, and result in polaron states, which activate the conductivity. PANI, in its basic form, is non-conductive. This doping procedure increases the conductivity of PANI via the removal of the quinone ring, electron cloud re-arrangement, and delocalization of positive charges on nitrogen atoms into a large conjugated π network [72]. While generally biocompatible, PANI’s alkali or salt derivatives have shown cytotoxicity, limiting its implementation in neural tissue engineering [79]. Another well-studied and reasonably priced CP with good biocompatibility is PPy. Applying PPy uniformly on electrospun nanofiber scaffolds increases axonal development and encourages cell differentiation [80].

#### 3.1.4. Natural Polymers

Collagen is the predominant protein in the extracellular matrix (ECM) and thus makes a very vital contribution to the structural integrity of the tissues. It follows that the materials chosen should have physical, chemical, and structural characteristics that help in neural repair. Inevitably, factors such as porosity, mechanical properties, biocompatibility, and biodegradation will have an enormous impact on the results. In NTE (neural tissue engineering), several types of biomaterials have been employed, ranging from synthetic polymers and polysaccharides to proteins. 

One of these proteins is collagen, mammals’ most prevalent protein. It constitutes about 25–30% of total protein. Studies have detected 28 types of collagens, each consisting of 3 polypeptide chains (α-chains) twisted together in a triple helix with the repeating motif of amino acids Gly-X-Y. Collagens I, II, and III have been reported to make up about 80–90% of the body’s collagen. Collagen I is present in skin, bones, and tendons, II in cartilage, and III in elastic tissues, such as lungs and blood vessels. This is because of the hierarchical structure of collagen, which makes it a highly adaptable and versatile molecule for tissue engineering [81,82,83]. With neuronal applications, collagen-based scaffolds offer a supportive matrix that enhances cell adhesion, migration, and differentiation. They can be custom designed to have specific mechanical and biochemical properties, and thus enhance neural regeneration [81].

Collagen is a leading natural biomaterial with significant potential for therapeutic applications in NTE, such as tissue repair and functional recovery. Collagen-based materials are used to address challenges such as neuroprotection and neuroregeneration. They can be converted into nerve conduits, scaffolds, injectable hydrogels, and microspheres. These forms must first offer structural support and create a supportive environment for neuronal growth; second, release growth factors to stimulate neural regeneration, especially in NDDs; third, encapsulate neural-related cells for therapeutic efficacy [83].

Recent developments in tissue engineering have made it possible to produce collagen scaffolds using different methods. Yannas et al. were pioneers in the freeze-drying prototyping of porous collagen-glycosaminoglycan scaffolds that could release chemicals therapeutically active during the degradation process [84]. Other hallmarks of scaffolds encompass thermal responsiveness, pH sensitivity, and regulated drug release at the site of injury [85]. This potential was further demonstrated in CNS repairs through the use of collagen scaffolds, which protect against inflammation, excitotoxicity, and oxidative stress, while also safeguarding cholinergic neurons in Alzheimer’s disease (AD), and by keeping toxic plaques out of the brain. While this treatment can work, it is not comprehensive in the treatment of spinal cord injury (SCI), and the isolated cells that are at the site of injury will eventually require physical bridging. This is exemplified by the development of an NSC-incorporated collagen scaffold that uses highly aligned, micro-channeled scaffolds to stimulate neural outgrowth and axonal reconnection in enhancing SCI repair. Researchers incorporated native collagen, de-hydrothermally cross-linked collagen implants with laminin, and epidermal growth factor receptor (EGFR) inhibitors to enhance an adult rat NSC regenerative process of neural progenitor cells (NPCs) toward SCI healing [83].

Collagen has recently received FDA clinical approval for use in peripheral nerve tissue repair, as it promotes nerve repair in a range of nerve scaffolds, such as NeuraGen, Neuromaix, and nerve cuffs NeuroWrap and NeuroMend, exhibiting degradation rates extending from months to years [86]. Likewise, this family of scaffolds can be biofunctionalized by growth factors or other proteins that further improve nerve repair. For example, nerve growth factor (NGF) combined with collagen scaffolding has been able to repair nerve gaps from 5 to 35 mm-long in several different animal models [87,88].

Therefore, it is a matter of selection of the most suitable cell sources for nerve regeneration. Common approaches for CNS regeneration involve transplanting neural stem cells (NSCs) or differentiated neural cells, along with integrating growth factors and glial cells to promote neuronal differentiation. The common cell sources for CNS regeneration include stem cells, such as induced pluripotent stem cells (iPSCs), embryonic stem cells (ES), NSCs, neural precursors cells (NPCs), oligodendrocyte progenitor cells (OPCs), MSCs, etc., wherein these are induced to differentiate specifically into the respective neuronal lineages for successful axonal outgrowth.

The main glial cells for the regeneration of the PNS are Schwann cells, with less significant roles played by astrocytes in conjunction with it and MSC. Schwann cells promote axon support in the enlistment of growth factors and in forming the myelin sheath. Extracellular components can be deposited by SC, such as laminin and type IV collagen, both of which assist in PNI repair.

Porous collagen-based scaffolds (PCS) have undoubtedly transformed the clinical management of peripheral nerve injuries. Despite it being 40 years since the discovery of PCS, it has not yet impacted the clinical management of CNS injuries and SCI [84]. Cell-free PCS grafts used on fully transected rat spinal cord injury models effectively reduced and favorably aligned collagenous scar tissue, leading to a decrease in cyst cavity formation at the lesion site [89]. When compared to cell-free grafts, NSC-seeded PCS grafts in the identical rat transaction SCI paradigm did not provide a discernible functional improvement. The difficulty of reversing full-transection SCI or the use of adult NSCs as opposed to embryonic ones may be the cause of this lack of improvement. Although there is already a great deal of research that reports on the effects of various biomaterials in NSCs, with only a few of them using porous scaffolds, the term “scaffold” in this work refers to a porous biomaterial manufactured in the dry state and, therefore, excludes hydrogels. Very often, these reports miss a thorough description of the used biomaterial, for example, structure, in vivo degradation, and surface-level chemicals, which rules out the possibility of drawing conclusions about which specific biomaterial characteristics have major in vivo regeneration effects in SCI [90]. No study has ever fully described PCS-mediated actions on potent NSCs, and the competency of PCS will provide a suitable support matrix for NSCs at SCI sites. A study investigated whether NSCs could in vivo engraft into PCS grafts and tap into the regenerative potential of NSCs for SCI, encouraged by the ways that fibrin hydrogels affect NSCs in vivo and how the keratinocyte-seeded Dermis Regeneration Template might hasten the development of epidermis in major skin lesions [91].

It was demonstrated that PCS grafts, manufactured from a well-characterized polymer similar to FDA-approved scaffolds used in regenerative medicine, can successfully deliver and preserve embryonic NSCs at the locations of spinal cord injury (SCI) to provide a statistically significant improvement in the recovery of locomotion in the mouse dorsal column crush SCI model, such that 12 weeks after the injury, the locomotion performance was identical to that of age-matched, undamaged controls [84]. An abundance of in vitro and in vivo evidence confirms PCS grafts’ capacity to regulate important functions that are necessary for the induction of regeneration in lesions of the CNS. In particular, the PCS grafts protected NSCs and supported NSC neural differentiation and functional integration in vivo. Furthermore, they promoted NSC migration into the surrounding tissue, enhanced axonal elongation at the lesion site and through the lesion boundary, and ultimately decreased astrogliosis. Our results support the creation of a novel class of SCI grafts that will distribute NSCs through enhanced scaffolds made from well-studied and clinically validated PCS [81].

One of the primary difficulties connected with NTE is the recreation of the native extracellular matrix. Cells in vivo are encircled by ECM, a dynamic three-dimensional network with collagen, proteoglycans, glycoproteins, and integrated bioactive ligands. It provides not only structural support but also modulates actions, such as proliferation, migration, and differentiation. Biomaterials based on collagen have discovered broad application in the repair of both CNS and PNS as scaffolds and hydrogels. Soft neural tissue is a soft matrix with mechanical properties, such as those of brain tissue, with the stiffness being ~0.5 kPa [92]. Therefore, therapeutic biomaterials should firstly biomechanically match the mechanical properties of neural tissue; secondly, host cell attachment, growth, and differentiation; thirdly, deliver bioactive substances that encourage the regeneration of axons; lastly, provide electrical conductivity to improve the development and differentiation of neural cells. Such requirements can be met using techniques such as nanotexturing, solvent casting, nonsolvent-induced phase separation (NIPS), and thermally induced phase separation (TIPS) [83,93]. 

There is a very high compliance exhibited by the brain and spinal cord, and thus the implants need to be compliant to match them and endure load forces. Ideal transplants maintain the structure and conformation to lesion cavities. Neurite development is enhanced on soft substrates, while astrocyte growth is favored on stiff ones. The NSCs preferred softer gels, about ~100–500 Pa, while the glial cells had a stiffer gel, ~1–10 kPa [94]. The tuning of neural implant compliance to the native tissue elicits minimal adverse responses, thus promoting biocompatibility. Recently, hydrogels have been engineered with specific values of native tissue for a given application, such as 400 kPa for spinal cord regeneration. 

Biochemical properties of scaffolds are important because of their interactions with cells. Functionalization of the scaffolds with neural-tissue-specific bioactive molecules, such as neurotrophin-3 (NT-3), fibroblast growth factor (FGF), basic fibroblast growth factor (bFGF), epidermal growth factor (EGF), brain-derived neurotrophic factor (BDNF), and glial-cell-line-derived neurotrophic factor (GDNF), enhances functionality. For example, in a study, guggulsterone-encapsulated drug-releasing microspheres were used in 3D bioprinted structures to promote neural development via bioink.

As electrical stimulation promotes neural regeneration, conductive polymers in scaffolds hold interest for NTE. Techniques include pre-blending and post-coating in order to achieve electrical conductivity. Polypyrrole has been utilized for its inclusion into scaffolds via inkjet printing or electrospinning to enhance neurite elongation, axonal regeneration, and remyelination [95,96]. While these in vitro studies are found quite common, utilization in clinics for electrical stimulation using conductive scaffolds was not successful. 

### 3.2. Therapeutic Opportunities: Neural Nerve Injuries and Tissue Engineering

The development of tissue engineering has revolutionized the treatment of neural nerve injuries, especially those related to the destruction of axonal tracts in the setting of PNI, SCI, TBI, and NDDs. Collagen, one of the major neural ECM constituents, has emerged as a very promising material for NTE. This section discusses the therapeutic uses of collagen in SCI, PNI, and TBI.

#### 3.2.1. Traumatic Brain Injury

TBI occurs due to external mechanical forces that induce short- and long-term damage to the brain. The natural healing processes are usually not adequate to heal the irregularly shaped lesions created by TBI and, therefore, require therapeutic interventions. Collagen, especially type I, has been used to treat brain injuries because of their biocompatibility with neural tissues and their ability to hold water as a hydrogel [83]. Studies have shown that collagen-based hydrogels loaded with stem cells or growth factors obviously promoted neuronal regeneration and functional recovery in TBI models [97,98]. 

#### 3.2.2. Spinal Cord Injury 

SCI can result in permanent motor and sensory dysfunction due to the complicated pathological processes involved. Collagen scaffolds have been applied in SCI repair for providing a supportive surrounding for the growth and development of cells. Functionalization of collagen with growth factors or bioactive substances has shown some potential applications in the improvement of axonal regrowth and decrease in glial scar formation [99]. State-of-the-art fabrication techniques, such as electrospinning combined with topological cues, have been created to improve the effectiveness of collagen scaffolds for SCI repair [99,100].

#### 3.2.3. Peripheral Nerve Defect Repair

While PNI readily leads to improved axonal regeneration compared to CNS lesions, the latter process is itself Schwann-cell-dependent. Collagen scaffolds are permissive to SC function and promote the regenerative response. Numerous collagen-based constructs, such as hydrogels, filaments, and films, have been fabricated that enable axonal growth and functional recovery in PNI models [101].

#### 3.2.4. Drug Screening and Disease Modeling

Traditional animal models of neurological disorders often lack translational potential to humans. Collagen-based hydrogels and, more so, 3D-bioprinted scaffolds, provide advanced platforms for drug screening and disease modeling that allow for specific investigations of cell–cell and cell–matrix interactions in a more physiological setting. For example, neuroblastoma has been modeled using 3D-bioprinted collagen constructs, as have other cancers, showing promise in precision medicine and chemotherapeutical evaluation [102,103].

Collagen-based biomaterials have shown great potential to be employed in neural tissue engineering treatments for TBI, SCI, and PNI since they can promote cell proliferation, differentiation, and axonal regeneration, besides the compatibility of these tissues with neural tissues for therapeutic applications. Moreover, collagen scaffolds could assist in modeling diseases and screening drugs to understand how cancer progresses and whether cancer treatment is effective. However, despite all this, research work should be continued and performed to optimize collagen implant functionality for a longer time of integration and functionality in neural tissue repair.

Because collagen is biocompatible, biodegradable, adaptable, reproducible, and low in immunogenicity, it is an ideal biomaterial in NTE. It is processed either as hydrogels for the repair of the damaged brain, as lyophilized scaffolds for SCIs and peripheral nerve injuries, or as enhanced hydrogels for drug testing and cancer modeling. Processing techniques for collagen-based biomaterials involve 3D bioprinting or electrospinning, and the resulting materials can be functionalized to build up a neural-friendly microenvironment [104]. 

Although some success in neural regeneration, PNI regeneration, and the study of neuropathology have been reported using the scaffold-free 3D bioprinting approach with the bioprinting technique, this method still has major limitations regarding bioprinting resolution and controlled delivery of bioactive factors compared to traditional scaffold-based methods [105]. The latter offers the biochemical signals and mechanical support required for neuronal development in restrictive settings. Even after impressive developments, the bench-to-bedside translation of collagen-based interventions is still difficult due to their expensive and laborious, standardized processes for neutralization and extraction. Potential avenues for future research in NTE include stem-cell-based gene therapy, drug screening, 3D bioprinting, brain–computer interfaces, and stimuli-responsive technology [83]. 

Post-hydration challenges in hydrogel formulations can lead to a decrease in mechanical strength. The sources of collagen are mainly tendons and skin of bovine and porcine origin and rat tail tendons. How far various sources lead to different cell behaviors or even diseases, such as BSE transmissions, needs to be studied. Allergic reactions to products from collagens of multiple species origins are also potential problems [106,107].

The clinical relevance of collagen extends to prognosis, diagnosis, and drug delivery for particular, targeted therapies [108]. Although preclinical studies are promising, the majority of collagen-based NTE applications remain preclinical, particularly for treatments of the CNS [109]. Clinical trials are ongoing concerning SCI repair, including safety and partial recovery of chronic SCI using MSC-loaded collagen scaffolds and the restoration of motor and sensory abilities of acute SCI. FDA-approved collagen-based nerve conduits, such as NeuraGen and Neuromaix, already demonstrated efficacy for PNS repair [110,111].

Further advancements aim to closely approximate the organization and microenvironment of native neural tissue by incorporating techniques that provide spatially patterned bioactive factors. The collagen-based biomaterials should ex vivo display relevant mechanical and biochemical properties for the brain and spinal cord, including viable cell types, such as OPCs, astrocytes, and microglia, in the context of CNS repair. Axonal alignment tools and long-distance lesion gap repair strategies are among the key technologies for PNI repair.

### 3.3. Gelatin in Neuronal Protection

Gelatin is denatured collagen that possesses most of the advantages of collagen but provides further advantages attributed to its special characteristics. Gelatin is an FDA-approved biomaterial that is known to be biocompatible, biodegradable, and low-antigenic, making it a good substitute for collagen in the construction of 3D hydrogels [112,113]. The cross-linking methods create porous gelatin hydrogels, which facilitate nutrient exchange and cell–matrix interactions and improve the alignment and adhesion of cells [114]. To promote neural stem cell development and neurite extension in neural engineering, gelatin hydrogels are mixed with polymers, including laminin, chitosan, and alginate [115,116,117]. However, 3D pure-gelatin hydrogels did not sustain neuron cell culture, which could be caused by an insufficient ratio of basic amino acids.

The amino acids in gelatin can communicate with pendant functional groups of all types through neural cells [118]. Cationic polymers promote neural cell adhesion and differentiation [119]. Lysine and arginine, contained in gelatin, may enhance the adhesion of neural cells. Though the function of lysine in neural behavior is known, that of arginine is not fully understood. As mentioned above, poly (allylguanidine) (PAG) had a more favorable impact on brain cells than poly-d-lysine (PDL); thus, it will be intriguing to find out if gelatin’s higher arginine content might improve neural activity.

A gelatin hydrogel with a somewhat elevated lysine content or guanidine group may have to be developed to study the functions of these amino acids. Poly-d-lysine is considered the gold standard for neuronal adhesion [120]. Neuronal adhesion and viability could be improved by previous investigations of poly (allylguanidine) [121]. Here, it was asked whether variations of lysine and arginine in the 3D gelatin hydrogel can guide neural cell responses, as applied in tissue engineering applications.

Hydrogels can be formed from cross-linked gelatin, offering a hydrated environment within which cells can survive and grow. This will yield a multifunctional platform for neural restoration by loading the hydrogel with drugs, growth factors, or even cells. The soft and flexible nature of the gelatin hydrogel makes it suitable for filling neural defects of irregular shape and providing support to regenerate tissues [82,122,123]. 

Neuron loss at the point of injury can stimulate glial cells to form glial scars that may restrict the damage but hinder axonal growth and neuron regeneration. One critical mission of nerve tissue engineering is the suppression of astrocyte over-proliferation and, hence, the formation of glial scars. Since amino acids included in gelatin aid in the control of cell behavior, it is a biocompatible biomaterial and a great matrix for tissue engineering. These biomaterials with a positive charge, such as PDL, encourage the attachment of brain cells. In this context, lysine and arginine residues present in gelatin will prompt the adhesion of neuronal cells and neurite outgrowth. The process of converting lysine’s ammonium into guanidine moieties enhanced neuron viability. Therefore, in this study, a 3D hydrogel of gelatin was created by using PAG to cross-link gelatin to increase guanidine content, along with PDL and polyethylenimine (PEI) for comparison [82].

The scaffold microarchitecture drives the neural cell response to the size of pores and internal topographies involved in guiding cell distribution and neuronal process alignment [124]. Maintenance of porosity in the gelatin-based hydrogels increased the potential cell contact and promoted neural process outgrowth [125].

In vitro and in vivo studies have assessed the effects of hydrogels on neuron survival and maturation through cell culture and animal studies. Alamar blue and Live/Dead assays revealed that only G-PAG hydrogel was able to improve the survival of neurons. Moreover, growth-associated protein 43 (GAP43) and synapsin I were linked to increased neurite outgrowth and neuron function, while anti-glial fibrillary acidic protein (GFAP)—a protein in astrocytes—decreased, enhancing neuron behavior and the inhibition of astrocyte survival [126]. Further studies in vivo revealed the efficacy of this G-PAG hydrogel in promoting axon expansion and inhibiting the growth of astrocytes, supporting its neural engineering potential [82].

The electrical characteristics, swelling ratio, and mechanical strength are related to the control of cell behavior [127]. Appropriate mechanical strength supports neuron survival and axonal outgrowth but prevents astrocyte proliferation [128]. The positively charged hydrogels enhance neuron attachment and differentiation [129]. The introduction of the polycations increased water absorption and softened the hydrogel structure, and it also increased the surface potential [127]. The differences in neural cell survival and neurite outgrowth were not caused by changes in hydrogel’s physical characteristics.

Moreover, chemical structure is essential for cell adhesion and processing. Increased guanidine in G-PAG supports guanidine’s role in neuron behavior regulation [130]. Results indicated that neural cell behavior depended on the polycation used. While the hydrophilic PDL and PEI backbones maintain extended conformations that result in neurotoxicity, the hydrophobic PAG backbone develops a globule conformation. Excessive adhesion to transforming growth factor beta (TGF-β) can trigger inflammation and cell death during the coil-to-globule transformation of the polymer [121]. PEI also induces reactive oxygen species (ROS), which mediates mitochondrial toxicity and leads to cell death. In contrast, the globule conformation of PAG will reduce the number of interactive proteins exposed to neural cells while G-PEI and G-PDL increase the protein adhesion to the hydrogel, increasing the risk of cell–protein interactions and cell death. The fact that neurons in 2D guanidine-modified gelatin or 3D PAG-modified gelatin hydrogel exhibited several branches and extended neurites demonstrated that the connections between neural cells and gelatin were heavily reliant on the guanidine moieties [82].

Altogether, collagen and gelatin play vital roles in neuronal regeneration and protection. Their biocompatibility and biodegradability, coupled with their ability to provide cellular functions, have made them more than essential in formulating higher-order therapies for neurological disorders. Research in this direction is continuing with the optimization of these natural polymers for better clinical outcomes.

### 3.4. Self-Assembling Peptide Nanoparticles 

Self-assembling peptide nanofibers offer a quite new and exciting opportunity in the field of neurology, with specific applications in brain repair and regeneration. The nanofibers can imitate natural ECM and provide a positive environment for neural growth and repair. The peptides are self-assembled; under physiological conditions, the nanostructure is defined by non-covalent interactions of the peptides. These interactions are majorly driven by hydrophobic contacts, electrostatic forces, and hydrogen bonds [16].

One of the massive advantages of peptide nanofibers self-assembling is that they can be easily functionalized to hold bioactive cues that will direct specific cellular responses. For example, these nanofibers could be functionalized with signaling molecules that will positively regulate neural stem cell migration, differentiation, and axonal growth [17]. This kind of functionalization plays an important role in enhancing the therapeutic potential of nanofibers for neurological disorders’ treatment [18]. In brain applications, it has been shown that self-assembling peptide nanofibers are very promising in several areas.

These nanofibers are capable of forming hydrogels that naturally mimic the brain’s ECM to provide structural support for regenerating neural tissues. The hydrogels could be engineered to undergo remodeling in response to specific biological signals, such as matrix metalloproteinases involved in tissue remodeling and repair [19]. For example, one study has illustrated the use of matrix metalloproteinase (MMP)-responsive hydrogel in supporting neural tissue regeneration through cell migration and axonal growth [20].

Peptide nanofibers have the potential to act as direct carriers for drugs within the brain. With altered nanofiber surface properties, it is possible to achieve targeted delivery for enhanced efficacy of therapeutic agents and reduced side effects [21]. Targeted delivery is of much more concern in the brain than in other organs because of the BBB, which poses problems to drug delivery [28]. This makes nanofibers quite useful for the creation of a biomimetic environment and, therefore, in the control of neural stem cell behavior, including the promotion of their migration toward injury sites, support of survival, and induction of differentiation into specific neural cell types [29]. This dynamic interaction between the nanofibers and the neural stem cells boosts regeneration to be more effective and efficient [30].

Such self-assembling peptide nanofibers can provide a scaffold for axonal growth, guiding the regenerating axons. Restoration of the integrity of neural networks is crucial for the recovery of function in disorders resulting from spinal cord injuries and neurodegenerative diseases [31]. There is evidence that such nanofiber scaffolds significantly enhance the outcomes of neural repair through the provision of relevant structural support and biochemical signals [32].

These nanofibers could further be functionalized into MMP-responsive hydrogels, thus making them an advanced tool in the neural repair realm. MMP-responsive hydrogels will help in dynamic communication between the neural tissue and themselves through self-destruction as a result of MMP activity, thereby releasing embedded therapeutic agents or making space for new tissue growth. This provides for dynamic changes to the environment within the brain and protection for tissue regeneration in a sustainable way [33]. In summary, self-assembling peptide nanofibers have huge potential in neurological and brain applications due to their ability to imitate the ECM, deliver drugs, modulate neural stem cells, and be conducive to axonal growth—all of which make them a versatile and powerful tool for brain repair and regeneration [34]. The process of continuous research and development can result in newer treatments against different neurological disorders, hence improving prospects in patients with brain injuries or neurological diseases [35].

## 4. Neural Regeneration

### 4.1. Neural Stem Cells

Strong self-renewal and the ability to develop into multiple neuronal and glial cell types are two characteristics of neural stem cells, a kind of multipotent cell found in all main divisions of the adult central nervous system, including the spinal cord [131,132]. In the presence of NSCs, the renewal potential for all cell populations of the CNS plays a very significant role in maintaining the functions of the CNS during development [133,134]. Self-renewal and generation of neural progenitor cells are characteristics unique to NSCs—they can self-renew. Moreover, they can differentiate into the following distinct mature neural lineages: oligodendrocytes, astrocytes, and neurons [135,136]. NSCs are spread throughout the whole axis of the CNS. The lateral ventricles’ subventricular zone and the hippocampus dentate’s sub-granular zone gyrus are two major areas in the brain that are actively involved in the renewal process of the neural stem cells [137,138]. 

Together with the astrocytes, oligodendrocytes are a major type of glial cells in the CNS. NSCs give rise to oligodendrocytes throughout the process of brain development, and their final phenotype is achieved through a series of differentiation processes. The final maturation of oligodendrocytes is critical for generating and maintaining the myelin sheath, a lipid-rich substance that envelops axons, providing electrical insulation and support. Therefore, the development of the oligodendrocyte lineage becomes important in myelination. In addition to facilitating quick electrical signal transmission down the axon, myelin provides neurons trophic support. In the brain and spinal cord, oligodendrocytes are found throughout the central nervous system (CNS) and are essential to the proper operation of the nervous system [132]. Numerous CNS illnesses, such as multiple sclerosis, acute disseminated encephalomyelitis, and other demyelinating disorders, might be linked to demyelination and neurodegeneration as a result of their loss or damage [139,140].

In many neurological disorders and injuries, it is the myelin sheath—the insulating layer surrounding the nerve fibers that ensures the effective transmission of electrical signals—that suffers damage [141]. Myelin regeneration is, hence, necessitated in the recovery process from such cases of damage. Remyelination, the regenerative response of myelin after demyelination, restores saltatory conduction and function, preserves the health of axons, and is necessary for the healing of demyelinating illnesses and neurological damage [142,143]. It has previously been demonstrated that NSC-derived oligodendrocytes may remyelinate injured nerve fibers and integrate into preexisting neural circuits [144].

The recruitment of NSC-derived oligodendrocyte precursor cells to the demyelination site is a complicated process that results in their differentiation into mature oligodendrocytes and the eventual formation of new myelin sheaths surrounding axons [145,146]. A network of transcription factors, signaling molecules, and microRNAs (miRNAs) controls the process [147,148,149,150,151].

Although NSC transplantation has been a very promising treatment for a variety of neurological diseases, several obstacles should be overcome before the wide diffusion of the therapy in clinical practice. These challenges include enhancing the survival and differentiation rates of the transplanted cells, guaranteeing the security and effectiveness of the administered treatments, and elucidating the mechanisms behind NSC transplantation-mediated therapeutic effects [152]. Long-term investigations must be directed toward cell source expansion, transplantation strategies, and streamlining preclinical animal models of human conditions.

In addition to cell transplantation, several NSC-based procedures have been developed. They include co-transplantation therapies, cell transport techniques, enrichment of the microenvironment, pharmacotherapeutics, biomaterial scaffolds, and neurorehabilitation, all of which greatly aid in improving brain repair [153,154]. 

In future perspectives, NSCs hold significant potential as transformative therapies for NDDs and SCI. As research advances, NSCs may offer innovative solutions for neuronal regeneration by differentiating into a range of neuronal and glial cell types, thus enabling the repair of damaged neural tissue and restoration of lost functions. With ongoing efforts to enhance NSC integration, survival, and differentiation, these cells could revolutionize treatment approaches, providing new avenues for reversing neurodegeneration and facilitating recovery from SCI [155]. The continued exploration of NSCs promises to unlock new therapies that could ultimately reshape the landscape of neurorehabilitation and functional recovery. There is every confidence that NSCs will soon come out with remarkable breakthroughs in the understanding of pathological mechanisms and the development of clinical treatments for various neural injuries and diseases.

### 4.2. Axonal Growth and Neural Stem Migration

The aligned nanofiber structure closely mimics the natural extracellular matrix, providing physical guidance cues that support neuron growth and alignment. Moreover, the integration of microparticles within the nanofiber yarns allowed for the controlled release of neuroprotective agents, which could help reduce oxidative stress and inflammation in neurons. These features suggest that the engineered nanofiber yarns not only serve as structural support but also actively contribute to neuron protection, enhancing their potential application in neural tissue engineering [156,157].

Zhang et al. detailed the fabrication of uniaxially aligned nanofiber yarns integrated with microparticles using electrospinning and electrospray techniques. The nanofiber yarns, primarily composed of biodegradable PCL, exhibited a highly aligned structure, with magnified views confirming their orientation [156]. Microparticles, spherical and rough-surfaced due to chloroform volatilization during electrospray, were successfully integrated into the nanofiber yarns. Temperature profiles during welding were analyzed under various laser conditions, demonstrating optimal conditions for effective fusion without structural loss [157]. Using these optimized parameters, the researchers successfully welded PCL nanofiber yarns with microparticles, maintaining their original morphology and structural integrity. Additionally, core-shell microparticles and varying deposition times were explored to modulate surface topology [156]. 

Further investigation focused on the release kinetics of bovine serum albumin (BSA) and anticancer drug doxorubicin (DOX) payloads from the microparticles welded onto the nanofiber yarns. Both payloads exhibited initial burst releases followed by sustained release patterns over 50 days, crucial for replicating native microenvironments and influencing neural cell behaviors [158]. The study also assessed the proliferation and morphology of SCs cultured on the microparticle-welded nanofiber yarns. Enhanced cell adhesion and alignment along the fiber direction were observed, highlighting the scaffold’s potential for nerve repair applications. Moreover, the study investigated axonal growth using PC12 and SH-SY5Y cells cultured on these scaffolds. Cells showed directional growth along the aligned nanofiber yarns, with enhanced viability and axonal length observed in microparticle-welded samples compared to controls [157]. 

Additionally, the migration of NSCs was studied on scaffolds with varying microparticle densities and NGF supplementation. Results indicated that both NGF’s biochemical signals and the microparticles’ topographic cues significantly influenced NSC migration, emphasizing the scaffold’s potential for nerve regeneration. Overall, the study demonstrated that integrating microparticles onto nanofiber yarns enhanced their functionality in guiding axonal growth and promoting cell migration, indicating their potential for use in brain tissue engineering and other associated biomedical applications [158]. 

## 5. Challenges in Delivering Therapeutics to the Brain: Overcoming the Blood–Brain Barrier for Effective Neural Regeneration

One of the greatest challenges facing medicinal and therapeutic provision today is delivering drugs and therapeutic agents across a very selectively acting blood–brain barrier. The BBB consists of tightly aligned endothelial cells, astrocyte end-feet, and pericytes, which come together to form a protective barrier, controlling and regulating substance migration from the brain to the blood [159]. This presents an important barrier to the microenvironment of the brain but poses a significant challenge to drug delivery by severely restricting the entry of most therapeutics, many small molecules, proteins, and gene therapies [160]. 

Since crossing the BBB is necessary for providing neural regeneration by delivering regenerative compounds directly to the site of injury or degeneration, it assumes importance. It is important for therapeutic agents to reach areas of damage within the brain and drive repair processes, including neurogenesis, remyelination, and synaptic plasticity [161]. Innovative strategies are being explored that transiently safely disrupt the BBB or exploit physiological transport mechanisms to enhance drug delivery [162]. These strategies involve nanoparticle utilization and changes in the drug itself. One challenge that must be considered in accomplishing this is the even greater one of the BBB, sitting at the heart of moving new treatments for neurodegenerative diseases and traumatic brain injuries toward clinical use and helping patients suffering from these devastating diseases.

A pharmaceutical technique was developed to address healthcare challenges using virus-free nanocarriers for the delivery of gene treatments for brain disorders, such as Alzheimer’s and Parkinson’s diseases, Huntington’s disease, stroke, and many others [163]. Inorganic NPs, such as gold and iron oxide, polymer-based NPs, carbon nanotubes, micro-/nano-emulsions, dendrimers, scaffolds, and drug-loaded nano-emulsions, have been suggested and explored for NCs [164,165]. They have several advantages over viral vectors for nanomedicine-based gene delivery methods, as follow: enhancing targeting specificity, extended circulation time, high loading capacity, controlled drug release, and reduced immunogenicity [166]. So far, symptomatic treatments have been the only feasible therapy for brain disorders because drugs could not cross the BBB [167]. Such shortcomings can be overcome with nanotechnology-based therapies due to their unique benefits [168]. Some forms of NCs have already been approved by the FDA to be used in combination with several commercially available drugs.

Liposomes have a spherical structure comprising a phospholipid bilayer with a water-soluble core of size 100–400 nm. This facilitates the crossing of macromolecules over the BBB by increasing the lipophilicity [169]. The therapeutic advantages of liposomal NPs include that they are commercially available and the ease of synthesis, and the simple encapsulation of therapeutic compounds enhances their solubility as therapeutic agents. Upon entering the brain, the liposomes start passive diffusion, which facilitates drug delivery [170]. To distribute Apolipoprotein E to the brain affected by AD, for example, a liposome carrier system altered with a mannose ligand and cell-penetrating peptides was applied. Functionalized liposomes can be said to safely and effectively transfer high gene concentrations into target tissues for treating Alzheimer’s disease [171].

Extensive research is underway to discover the potential of polymeric nanoparticles ranging from 1 to 100 nm for the use of tailored medication delivery to treat brain gliomas [56]. Glioblastoma multiforme and other malignant brain gliomas might be treated with intra-tumoral administration of anticancer medications employing NPs [172]. Convection-enhanced delivery and NP encapsulation improved medication penetration, distribution, and survival in glioblastoma (GBM)-affected rats [166]. For example, Dox was injected intravenously into poly(butyl cyanoacrylate) NPs, which led to an enhanced amount of drug accumulation at the tumor site of glioma in rats [173]. Because polymeric NPs are both biocompatible and biodegradable, they represent a particularly novel approach to medication administration for non-disabled patients [174]. In the treatment of AD, recent research has concentrated on the use of curcumin-loaded PLGA NPs to improve medication delivery and lower oxidative stress and inflammation. In transgenic AD mice, functionalized polymeric biodegradable NPs containing an antibody and polyethylene glycol (PEG) have been demonstrated to ameliorate memory impairment and drastically lower levels of soluble A-peptide.

Dendrimers are nanosized, very definite formulations that offer the potential for treating neurodegenerative diseases. To create brain-delivered drug and gene nanocarriers, dendrimers’ size, core-shell configuration, and surface functional groups can be changed [166]. Dendrimers show anti-amyloidogenic activity and, hence, can be utilized in the treatment of prions, Parkinson’s disease (PD), and AD [175]. Phosphorus-containing dendrimers have been studied for their potential use in inhibiting prion infection because they can prevent misfolded prions that lead to a lot of brain diseases [176]. Dendrimers are nanoscale molecules that have a high degree of branching with well-defined structures, which enables perfect control over their properties and the addition of phosphorus to inhibit prion infection [177]. However, high dendrimer production costs are still a major drawback, and further research is needed into the health consequences of long-term exposure to dendrimers [178].

Micro/nano-emulsions have been designed with a hydrophobic core structure of 10–100 nm in diameter while being composed of block copolymers containing a hydrophilic shell. Such structures could contribute to the controlled release of an anticancer medication by boosting its solubility, enhancing its pharmacokinetics, and preventing the drug-conjugated core from interacting with the phagocytosis-complement cascade [179]. This shell architecture makes it perfectly suitable for PEG, which does not bind to serum proteins [180]. Micro/nano-formulations can induce target cells for tau protein degradation and activation of autophagy, which appears useful in treating NDDs. For instance, curcumin-loaded polymeric nano-formulations and glycated bovine serum albumin in phosphate-buffered saline may lessen the production of amyloid in AD mice [181].

Among the best inorganic metal NCs applied for the treatment of high-grade gliomas (HGG), gold nanoparticles have shown excellent results, thanks to their increased surface-to-volume ratio, ability to cross the blood–brain barrier, and size adjustability [182]. Nevertheless, AuNPs’ possible use in the treatment of HGG is still low since they cannot target only tumors. Recent developments in trapping AuNPs via gold–sulfur interactions improved the efficiency of a DNA aptamer targeting the expression of EGFRvIII in GBMs. They showed great promise in vivo and in vitro for the therapy of this disease [183]. By overcoming medication resistance, AuNPs have demonstrated enhanced drug accumulation in cancer therapy and assist in delivering targeted therapeutic genes [184,185].

The nano-emulsions have a size range of 10–1000 nm and possess special features for drug delivery, including a large capacity for transporting pharmaceuticals, stability, regulated drug release, excellent selectivity in focusing on certain cells or tissues, and ease of hydrophilic and hydrophobic chemical transportation. Such benefits overcome diverse types of issues that are often connected to traditional medication administration [186]. The delivery of NPs laden with drugs to the intended area could be caused by various mechanisms, including simple diffusion, degradation, erosion, and energy input from external sources [187]. AuNPs, with their flexible platform, exhibited improved drug accumulation in treating cancer treatment and showed an improved establishment over multidrug resistance [184].

Carbon nanotubes (CNTs), having superior physical and mechanical properties combined with a high aspect ratio and nanoscale size of less than 100 nm, profoundly affect the way medicinal substances are internalized by cells. Functionalization of the CNTs involves strategies related to the linking of a carboxyl group following oxidation, and an organic group attached to either the sidewall or the tip of the CNT. Advanced biocompatibility in polymer- and dendrimer-linked CNTs ensued with increased solubility and reduced aggregation [166]. While single-wall carbon nanotubes infused with acetylcholine have been investigated for the treatment of AD, and CNTs coupled with stem cell therapy have been used in stroke treatment, very few studies related to CNTs in the treatment of CNS disorders have been reported. Carbon nano-horns and nanodiamonds have been functionalized to increase the applications of nanotechnology in the biosciences and pharmaceutical industry [188,189].

## 6. Nanoparticles for BBB Modulation

### Nanomaterial Strategies for Crossing the BBB

The BBB, blood–cerebrospinal fluid barrier, and the arachnoid barrier constitute the critical interfaces that make up the distinctive cellular structure controlling the movements of various substances into the CNS. The BBB itself, composed of microvascular endothelial cells, is the largest interface of blood–brain exchange, with a total surface area of 12 to 18 m^2^ and a micro-vessel surface area of 150 to 200 cm^2^ [190]. It presents a critical barrier to the entry of pathogens into the brain and limits the entry of drugs and other exogenous molecules.

**Figure 2:**
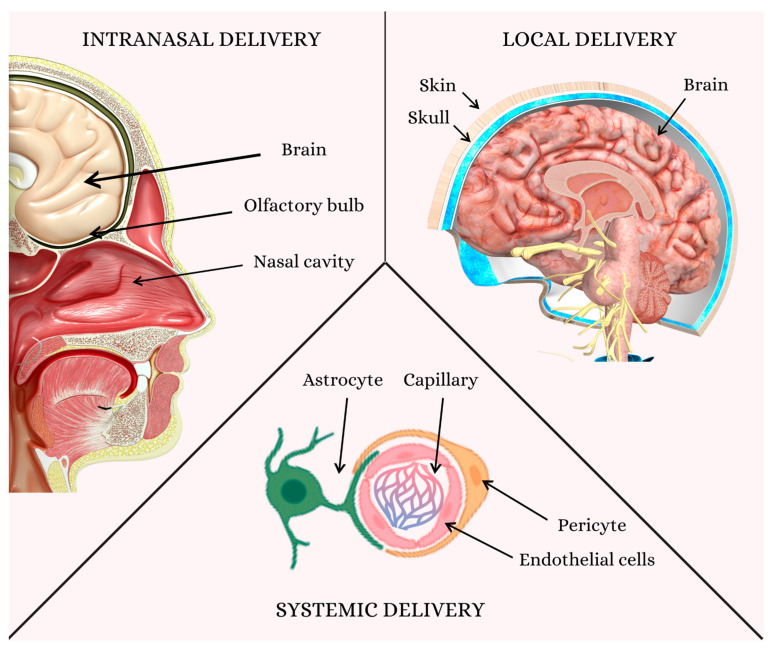
Methods of central nervous system drug administration.

Three primary approaches have been established for crossing the BBB: noninvasive, invasive, and alternative drug-delivery approaches [191]. The former includes mucosal or ocular delivery, such as intranasal delivery, which relies on uptake by the olfactory or trigeminal nerves, which bring the NPs straight into the brain, bypassing the BBB [192]. Although this method works well with some drugs, it has its limits when the proteins are bigger. It has been revealed that cell-penetrating peptides (CPPs) have proven to be a very promising tool for the delivery of macromolecules through cellular membranes with low toxicity [193]. The second, the invasive approach, is the temporary opening of the BBB to infuse drugs directly into the brain. Such techniques as osmotic disruption and FUS open the BBB transiently, allowing drugs to pass through the BBB itself, and direct administration of the medication while reducing the likelihood of systemic toxicity and safeguarding the surrounding healthy tissue. As such, the invasive method is far from ideal because it requires hospitalization, carries the risk of brain tissue scarring, and may lead to infections [194,195,196,197]. While these techniques increase therapeutic agent delivery, they can also result in inflammation or tissue damage risks [198]. Moreover, other methods, such as receptor-mediated transport or shuttle-peptide-mediated delivery, avoid permanent disruption of the BBB. These methods exploit specific ligand–receptor interactions that are expressed on endothelial cells to promote the delivery of therapeutic agents into the CNS (Figure 2) [191].

Some of the major advantages of NPs in medical delivery include extended bloodstream circulation time, controlled release of drugs, increased stability, a substantial dosage, and focused administration. There has been much research on these nanomaterials’ ability to transport medicinal drugs across the blood–brain barrier [199]. For example, the in vivo fate of nanomaterials is heavily influenced by their size, zeta potential, and hydrophilicity [200]. Most of the strategies that involve crossing the BBB using nanomaterials, as well as methods against neurodegenerative diseases, such as Alzheimer’s and Parkinson’s, have been significantly hampered by the very constraining characteristics of the BBB [201]. Nanomaterials have succeeded in enabling the transportation of pharmaceutical agents across the BBB using various drug-delivery methods [202].

Intranasal drug delivery is a productive, noninvasive approach to reach the brain with drugs after overriding a series of challenges of parenteral administration. Drugs can be effectively delivered to the brain via an intranasal administration procedure through the olfactory mucosa. This might involve diffusion across the connective tissue around the bundle of olfactory nerves or the axons of olfactory nerves, by which the drugs escape the tendency of crossing the BBB [203]. Drug buildup in non-target organs has been reduced and systemic adverse effects have been lessened by intranasal delivery, which has circumvented hepatic first-pass metabolism [204]. Intranasal delivery has also been a key route of mucosal delivery in pharmaceutical applications due to its advantages of rapid absorption, active onset of action, noninvasiveness, low tissue damage, and ease of application [193]. From this study, it was indicated that there were many variations in the medication delivery effectiveness of different intranasal delivery nanoparticles. The physicochemical characteristics of the medications that were administered had little bearing on these variations [205].

Extensive research has been carried out on the temporary opening of the BBB as the best method for enhancing the transport of drugs from the bloodstream into brain pathological conditions [206]. BBB manipulation mainly involves osmotic, ultrasonic, and magnetic disruption techniques [201]. Different pharmacological drugs have been locally delivered by many researchers who have studied the direct delivery of drugs in local brain disorders. Several treatments using locally delivered different pharmacological drugs have been developed. Early investigations have implanted biodegradable polymer wafers containing a pharmacological payload, enabling local non-pharmacological administration to the central nervous system [207]. The wafers release drugs in a controlled way for a long period, which provides better therapeutic responses.

Cationic CPPs are short and amphipathic [208]. It has been found that several peptides make conjugated medications or biomaterials more effective at translocating cell membranes. A pair of primary categories of cationic CPPs are chimeric peptides and antimicrobial sequences. Brain endothelial cell membrane surfaces and the cationic charges on CPPs are thought to interact electrostatically to facilitate the transport of CPP-modified nanomaterials across the blood–brain barrier (BBB), even though the precise mechanism by which CPPs promote transportation across cell membranes is unknown [166].

Numerous endogenous macromolecules are transported into the brain through the receptor-mediated transcytosis pathway [209]. Brain endothelial cells (BECs) are surface receptors for ligands that attach to them specifically. This process results in the creation of vesicles through the endocytosis pathway [210]. These vesicles release the ligands and, utilizing exocytosis, allow passage across the BBB and into the CNS, where they can execute their biological functions [211].

The interest in shuttle peptides is growing because of their inexpensive, widely available, less immunogenic, and highly chemical flexibility since they can resist chemical modifications [212]. Shuttle peptides can carry a variety of payloads across the BBB, including small molecules, genetic materials, proteins, and nanoparticles. Several peptides to shuttle the BBB have been constructed to load NPs, which boosts their delivery into the CNS [213]. 

## 7. Combining BBB Modulation with Neural Regeneration

Recent advancements in neurotherapeutics have focused on the innovative approach of combining blood–brain barrier (BBB) modulation with neural regeneration to enhance the delivery and efficacy of therapeutic agents [214]. This approach does learn to use BBB-modulating nanoparticles for the efficient deliverance of growth factors, stem cells, and drugs into the CNS to overcome one of the major hurdles in treating neurological diseases. These nanoparticles have other functions in the crossing of the BBB, which is of importance in neural repair and regeneration [215]. This provides an approach for several CNS diseases: traumatic brain injury, neurodegenerative diseases, and others, with in-depth analysis of promising results at both preclinical and clinical levels. Researchers have combined advanced systems of delivery with regenerative medicine in the hope of transforming treatment for a host of neurological conditions [12]. 

### 7.1. Neural Stem-Cell-Derived Exosomes

Exosomes are 40–100 nm phospholipid bilayer structures that are secreted by almost all mammalian cells. Once considered cellular waste, they are now recognized for their role in cell communication [216,217]. They can carry lipids, proteins, and nucleic acids and, further, facilitate long-distance communication, genetic exchange, and immune signaling. They even act as independent metabolic units [218,219]. They play a central role in immune response [220,221], apoptosis [222,223], angiogenesis [224,225], and inflammation [226]; therefore, they are also very promising for cell-based therapies, diagnostics, and drug delivery.

Brain injury repair is challenging due to the failure of neuron regeneration and the inhibitory environment resulting from TBI. Thus, strategies are focused on promoting neuroregeneration and creating an assistive environment. In this regard, evidence implies that the therapy of traumatic brain injury (TBI) may benefit more from exosomes made from stem cells than from direct stem cell transplantation [227,228]. This has opened new therapeutic avenues toward nerve repair [228]. Since Johnstone first described exosomes in 1987, their potential for use in regenerative medicine, especially regarding brain repair, has been gradually realized [229]. Exosomes play a significant role in intercellular communication within the brain and spread signals throughout the brain via cerebrospinal fluid [230].

Exosomes obtained from sources such as MSCs represent a new approach to precision medicine against neurodegenerative diseases [231]. After stem cell treatment, exosome therapy is a relatively new and exciting area of study. Neural stem cell exosomes provide neuroregulatory and reparative capabilities and participate in the pathological and physiological alterations associated with traumatic brain injury. Compared to the parent stem cells, exosomes are more stable, immunotolerant, and have less tumorigenic risk. The added advantage is that they cross the blood–brain barrier, which offers better efficiency for treatment. Their regenerative and immunomodulatory potential places neural stem-cell-derived exosomes as front-runners in the repair of TBI [232]. 

It has been reported that NSCs can improve neurological function through vascular and neuroregeneration stimulation. NSCs are derived from endogenous sources in the subventricular zone and the dentate gyrus or exogenous embryonic tissues. There are problems with both sources: exogenous NSCs may cause transplant reactions, and endogenous NSCs have low survival and/or differentiation problems [233]. 

Drawing from the mentioned challenges, researchers are searching for possible alternatives that could reproduce the NSC effects. There is a report that the beneficial effects of NSCs are due, to a large extent, to their paracrine signaling, especially via the release of neurotrophic factors and exosomes [234,235]. Among others, NSC exosomes have been described as the real player in cell communication and repair processes, being considered a potential new therapeutic approach to neurological diseases, enhancing neuroplasticity and recovery after injury [236]. It is believed that exosomes secreted by NSCs amplify the benefits of SC-based therapies via the suppression of risks and issues associated with the application of whole NSCs. It is hypothesized that NSC exosomes will readily permeate the blood–brain barrier, lower inflammation, block apoptosis, alter immunological responses, and control autophagy. As such, NSC exosomes represent a practically inexhaustible source of stem-cell-free therapeutic agents that hold great promise for TBI treatment, since they do possess all those features that a repair and regenerative therapy is supposed to have [232].

The interactions between neurons and microglia in the central nervous system are greatly influenced by exosomes derived from NSCs [237]. They can transmit active molecules to astrocytes, and thus regulate their activity, synaptic activity, and neurovascular integrity. While NSC exosomes may convey some pathological particles participating in some neurodegenerative disorders, including Alzheimer’s, prion, and Parkinson’s, they also exert neuroprotection, supporting nerve regeneration and repair.

These exosomes are enriched in specific miRNAs involved in several CNS disorders, intercellular transmission, and modulation of virus entry; additionally, they can function as separate metabolic units [238]. Exosomes derived from NSCs exhibit the expression of 113 human miRNAs, and some of them, such as miR-4488, are involved in regenerative functions. Thus, NSC exosomes can exert their functions in intercellular communication through the influencing of target cells via the interferon-gamma pathway. They are also able to deliver viruses to cells lacking specific receptors and alter the local nutrient environment. The exosomes from mouse NSCs are also enriched in miRNAs regulating the process of microglia’s growth and function of nervous system regeneration [232].

#### 7.1.1. Understanding NSC Exosomes in Brain Injury Regeneration

It has been previously demonstrated that neural stem cells have great potential for treating several neurological disorders, such as TBI, using experimental and clinical strategies [239,240]. Recent reports indicate that the main mechanisms underlying NSC activity related to brain remodeling and recovery could be independent of cell replacement and instead involve paracrine effects—including the exosomes they release [241]. These exosomes, originating from NSCs, package signaling molecules facilitating neuronal communication and aiding brain repair through the regulation of cell interactions. Investigations into the exosome contents from human iPSC-derived NSCs have revealed an enrichment of miRNAs and proteins promoting neuroregeneration, preventing apoptosis, reducing inflammation, and repairing the blood–brain barrier. They also promote synaptic plasticity and recovery of cognitive function. These changes in pathophysiology, rooted in various types of brain injuries and disorders, lead to acute and delayed neuronal damage, largely caused by programmed cell death [232].

Together with NSCs, the brain’s neurovascular units—which are made up of neurons, astrocytes, microglia, endothelial cells, perivascular cells, basement membrane, and extracellular matrix—assist in promoting the body’s natural healing processes, such as neurogenesis and vascular regeneration [242,243]. NSC-derived exosomes play a very significant role as signaling agents in supporting these repair processes. For example, circular RNA (circRNA) Acbd6 from hippocampal NSC exosomes has a beneficial modulatory effect on NSCs’ differentiation into cholinergic neurons via the miR-320-5p/oxysterol-binding protein-related protein 2 axis [244]. NSC exosomes have been shown to augment new neurons in the hippocampus, promote the proliferation of cells, and enhance cell vitality. Moreover, exosomal microRNA-210 (miR-210) from hypoxia-induced neural progenitor cells contributes to cell proliferation, while in other works, the role of exosomes in NSC differentiation was underlined, modulating the process via the miR-9-Hes1 axis [245,246].

Exosomes from NSC participate in multiple interactions with neurons and microglia to accomplish reparative tasks in the brain under various conditions [237]. They transmit active molecules to astrocytes, controlling the activity of astrocytes and synaptic functions, neurovascular integrity, and myelination. It has been revealed that exosomes derived from human NSC could lead to BBB leakage and promote the regeneration of nerve cells [247]. Exosomes derived from hiPSC-NSCs have been reported to increase hippocampal neurogenesis when administered intravenously by promoting exosome uptake by neurons and neuroglial cells throughout the adult rat brain [232]. This innovative approach opens new avenues for therapeutic delivery by harnessing the body’s natural pathways. Notably, the noninvasive method of intranasal administration has emerged as a promising route for delivering exosomes directly to the brain, bypassing the BBB, and enhancing the treatment of neurological disorders through improved neurogenesis and repair mechanisms.

Treatment applications of such central nervous system injury using exosomes is promising because of their unique nanostructure, low immunogenicity, minimal toxicity, and the capability to cross the BBB [248,249,250,251]. They are essential for tissue regeneration and immune regulation; however, they are often devoid of precise targeting. Engineered exosomes aid in the overcoming of this intrinsic limitation by actively targeting specific cells or tissues [252]. These appear to provide neuroprotective agents across CNS barriers to specific brain regions after newer advances in nanotechnology.

One of the key methods in the production of engineered exosomes is cell engineering. This approach involves genetic manipulation in the parent cells to alter the constituents of the exosomes at the point of formation. For instance, Alvarez-Erviti et al. demonstrated that exosomes engineered from dendritic cells with neuron-specific proteins could deliver siRNA into the brain with good efficiency [253]. On the other hand, drug incubation incorporates drugs, such as paclitaxel, into exosomes to enable specific targeting of, for instance, pancreatic cancer [254]. On top of the above, exosome engineering involves the manipulation of purified exosomes through methods such as incubation, physical methods, saponification, and surface modification, which have advantages regarding production efficiency and scalability [255,256,257].

NSC-derived, engineered exosomes for delivering specific miRNAs, mRNAs, and proteins have huge potential in treating brain injuries. Targeting the engineered exosomes to neurons for delivering therapeutic agents is possible. In a recent article, Chen et al. showed that exosomes loaded with BDNF increased neural stem cell survival and promoted their differentiation into neurons, and such exosomes also reduced inflammation and cerebral infarction in an ischemic stroke model [258]. Other studies have also shown that NSC-derived exosomes are capable of effectively crossing the BBB and delivering therapeutic cargo: fluorescent proteins in exosomes have been detected across the BBB, indicating drug-delivery competency [259]. It is possible to engineer NSCs to produce therapeutic exosomes continuously using booster plasmids, providing sustained treatment after intracerebral implantation [260].

#### 7.1.2. Challenges and Opportunities

There are many advantages of using neural stem cell-derived exosomes in treating brain injury. First, owing to the nanoscale size, exosomes could easily penetrate the BBB and be easily absorbed without the risk of occluding small vessels when injected intravenously [261]. Second, NSC-derived exosomes facilitate cell-to-cell communication, assisting in the conveyance of repair-related signals between the different cells in the damaged brain. Thirdly, they are lowly immunogenic, and thus may become a cell-free promising therapy for neurological diseases with a low level of side effects [262]. Finally, in contrast to stem cells, exosomes are more easily stored and processed to prevent several problems linked with the culturing of stem cells and the possible formation of tumors from them. Hence, understanding the role and mechanism of NSC exosomes in the process of brain injury repair can improve cell-based therapies.

Despite their potential, translating NSC-derived exosomes into clinical practice for brain injury treatment faces several challenges. These include the development of a stable system for NSC exosome production, standardization of purification and storage methods, and single-exosome analysis to better understand exosome-mediated regenerative mechanisms [263]. Exosomes can display a high degree of heterogeneity since they are small particles with variable protein composition and changes in gene expression driven by a host of factors, from circadian rhythm to stress and infection [264]. Efficient purification methods are, therefore, required to obtain a homogeneous population of NSC exosomes that incorporate similar RNA, proteins, and lipids. Most currently available methods have been less than satisfactory, with either poor yield or quality or in retaining the activity of the exosome through storage [263]. Thus, the two techniques should be developed for the progress of NSC exosome therapy in brain injuries.

Also, although NSC exosomes seem to be a very promising means of treatment, more information is needed about their biological activities and detailed mechanisms of interaction with target cells. Advanced imaging techniques can track the release, transport process, and fate of NSC exosomes at a single-vesicle level, providing further information about their functions to promote further clinical application. Moreover, they could be complexed with other drug-loaded NPs, which may extend their therapeutic potential and make them more promising therapeutic agents [232].

### 7.2. Neuromodulation-Based Stem Cell Therapy

Changes in the stem cell method for brain healing can be made by either promoting the migration and proliferation of stem cells to support endogenous neurogenesis or by transplanting exogenous stem cells either directly to the site of injury or through the bloodstream. Neural stem cells have been targeted in clinical trials for the treatment of Parkinson’s disease, amyotrophic lateral sclerosis (ALS), and ischemic stroke [265,266]. However, the challenges with this approach are based on the inability to reform functional neural circuits at the site of damage. Additionally, the actual differentiation and maturation of specific cell lineages also present further challenges. To successfully apply stem cell therapy, mature transplanted cells must replace the degenerated host neurons and project patterns to reform and reconstruct precise synaptic transmission [266]. Most of the time, engrafted neurons are not able to integrate themselves precisely into host neural circuits, and this can lead to variable functional outcomes and serious side effects. Incorrect projection patterns from grafted neurons can lead to the disruption of function. 

Strategies needed to foster functional recovery involve a range of complementary options for improving the differentiation and maturation of stem cells. The invasiveness or noninvasiveness of neuromodulation techniques varies, and there are many such techniques: deep brain stimulation (DBS), transcranial magnetic stimulation, transcranial direct current stimulation, and transcranial ultrasound stimulation. Stem cell neuromodulation deals with bolstering the differentiation, maturation process, and functional integration of grafted neurons. The next sections appraise the evidence that neuromodulation has opened the doors that someday might be a promising strategy for its augmentation to strengthen the efficacy of stem cell therapy [267].

#### Neuromodulation

One of the most important issues that needs to be resolved is gaining a thorough grasp of the therapeutic processes of DBS, above all for investigative indications. Early ideas included the decoupling of the axon and soma, which has been variably defined as an informational lesion, and the local suppression of pathogenic activity in the targeted area by the progression model in Parkinson’s disease [268]. A pulse generator that is implanted in the chest behind the clavicle is connected to one or more electrode leads that are inserted into the brain parenchyma via extension wires that pass beneath the skin. The DBS system typically uses a continuous current > 100 Hz in frequency [269]. It has been suggested that high-frequency (HF) stimulation is too rapid for excitatory neurons to follow through. Therefore, the regional response to the HF DBS is predominantly through the activation of the inhibitory networks. Pallidothalamic, cerebellothalamic, and pallidonigral fiber networks, for instance, all contribute to the same therapeutic response to the DBS, and stimulation of the subthalamic nucleus (STN) can activate nigrostriatal by increasing dopamine release [270]. 

The DBS lead location is additionally a critical factor for postsurgical outcomes, and how well the choice and targeting of networks occur in DBS is key for optimal outcomes [271]. Surgical planning usually uses a combination of magnetic resonance imaging (MRI) and stereotactic-atlas-based targeting. Intraoperatively, microelectrode array recordings can be used to map the borders of a target region, and postoperative imaging can be used to verify electrode placement [272,273]. Computational models link the stimulation area to patient outcomes, establishing a “sweet spot” within target brain regions [271,274]. Computational modeling has similarly been utilized to explain single-neuron modulation as a product of stimulation and synaptic plasticity [275]. The amount of research is large, and it converges on a single, cohesive theory: DBS operates via a multimodal mechanism [276]. One prominent theory suggests that DBS’s therapeutic effects result from adjusting activity across target networks [276,277]. This is in line with the observation that many diseases for which DBS is used have been described as network disorders, rendering DBS a network therapy [278,279]. Identification of target networks, therefore, seems basic to the optimization of outcomes from DBS.

Research has shown that the primary mechanism by which STN DBS affects critical tremors is via activating the cerebellothalamic fiber networks that run immediately posterior and ventral to the STN. For this reason, DBS targeting the STN, or globus pallidus internus, is frequently used to stimulate motor function and balance in patients with late-stage Parkinson’s disease. Factors that contribute to the neural mechanism by which the effects of DBS on PD provide therapeutic gains include improved dopamine release, regional oscillation, restored balance of excitation/inhibition, and the normalization of neural network connectivity [267,280].

The idea that aberrant network oscillations cause various neuropsychiatric symptoms treatable by DBS and motor dysfunction in movement disorders is becoming more widely accepted [268]. Network-based biomarkers of DBS clinical response could identify therapeutic stimulation parameters. Advances in DBS technology have now enabled whole-brain 1.5 T or 3 T MRI of patients on stimulation, so functional magnetic resonance imaging (fMRI) shows itself as a compelling method for the in vivo investigation of the consequences of whole-brain DBS [281,282,283]. 

Two main causes of variability are the fMRI paradigm and the time of the imaging. Following DBS parameter optimization, the results are susceptible to both acute and chronic stimulation effects [284]. Continuous cycling stimulation during rest, or comparisons at rest versus during tasks performance in DBS on and off states, are common fMRI paradigms. These paradigms shed light on the impact of stimulation on functional connectivity as well as the task- and DBS-evoked Blood Oxygen Level-Dependent (BOLD) responses. The effects of DBS on brain function have generally proven repeatable, notwithstanding these variations. For instance, the cerebellum’s and the cortico-basal ganglia-thalamo-cortical network’s BOLD activity have been consistently altered in Parkinson’s disease (PD) by cycling STN DBS. These outcomes have been linked to an improvement in Parkinson’s disease motor symptoms with DBS, paving the way for the use of fMRI as a therapeutic programming tool [285].

Since globus pallidus pars interna (GPi) is now known to be a target for dystonia and Parkinson’s disease (PD), current research has concentrated on figuring out how GPi-DBS affects motor networks [286,287]. Evidence of corticothalamic connection alterations and GPi-DBS-evoked brain network normalization overlaps with STN-DBS modulatory effects, suggesting commonalities across diseases of basal ganglia dysfunction treated at the same site. Those types of approaches are only beginning to emerge for the DBS management of neuropsychiatric conditions. For these disorders, fMRI presents itself as a very promising technique to elucidate time courses of DBS effects and to determine efficient stimulation parameters with little patient feedback [268,288]. 

Therapeutic stimulation settings reduce BOLD activity in regions compatible with the default mode network, and there is a corresponding drop in the connection between the anterior limb of the internal capsule (ACC) and frontal areas, according to a recent fMRI study of OCD patients treated with DBS to the internal capsule. Longitudinal analysis revealed reductions in the connectivity of the ACC-striatal and regional BOLD variability, indicating the neuroplastic effects of DBS. This will call for further multimodal studies in larger cohorts, leading to improved clinical outcomes and enhanced understanding of DBS circuit engagement. This roundtable discussion on sweet-spot mapping and fMRI brings to light techniques for the advancement of DBS clinical outcomes through lead localization and the optimization of stimulation parameters [289,290,291,292,293,294,295]. More intergroup collaboration, further database establishment, and sharing for more common DBS indications would even more significantly enhance the knowledge base and practice. Future research should investigate the causal links between applications and treatment results across targeted networks by integrating these data into multimodal analysis.

### 7.3. Mesenchymal Stem Cells (MSCs)

In the past few years, stem cell therapy has moved beyond its classic indications for hematopoietic disorders and includes a much larger spectrum of diseases. Among these, MSCs have emerged as one of the most prominent cells attracting attention for neuronal regenerative medicine. It is well known that MSCs have the potential for pluripotency—meaning, the capability to differentiate along multiple lineages—with a high self-renewal capacity and immunomodulatory effects. Of particular interest, these features make MSCs very promising for the repair of damaged neural tissues and support of neuronal function regeneration [296,297,298,299,300,301,302,303]. MSCs are emerging as a powerful asset in revolutionizing treatments for neurodegenerative diseases and brain injuries, offering renewed prospects for effective therapeutic solutions.

In the late 1960s, Friedenstein and colleagues highlighted mesenchymal stem cells as multipotent stem cells capable of differentiating into mesodermal (adipocytes, osteocytes, and chondrocytes), ectodermal (neurocytes), and endodermal (hepatocytes) lineages [304,305,306]. Initially considered “stromal” cells, MSCs have been recognized for their regenerative potential rather than their stem cell status [307]. They can release prostaglandin E2 (PGE2), which is crucial for their self-renewal and regenerative capabilities [308]. 

Primarily found in bone marrow, MSCs can also be sourced from tissues, such as adipose tissue, umbilical cord, and dental pulp [309,310,311]. They are characterized by markers, such as CD73, CD105, and CD90, and they lack markers associated with hematopoietic cells [312]. MSCs have shown significant capacity to differentiate into several cell types, giving them promise in regenerative medicine, supporting tissue repair, and releasing bioactive components, such as growth factors and cytokines [313,314]. Their ease of isolation, rapid expansion, and low risk of teratoma formation make them a valuable resource for regenerative therapies [315].

#### Role of MSCs in Neuronal Regeneration

Numerous neurological conditions have previously been demonstrated to respond well to the use of bone marrow stem cells (BMSCs). In the case of ALS, intrathecal administration of autologous BMSCs was reportedly associated with improvement in Amyotrophic Lateral Sclerosis Functional Rating Scale (ALSFRS) scores and stabilization of forced vital capacity (FVC) in 75% of patients at two years—perhaps indicative of a slowing of the progression of the disease. The results, however, have been quite mixed. Improvements were witnessed with Wharton’s jelly MSCs by Barczewska et al., while a study with autologous adipose MSCs reported limited benefits [316,317]. ALSFRS-Revised scores were stable, and the levels of TGF-β and IL-10 were improved, according to OH et al. However, generalization was limited because of the small sample size [318].

Some potential benefits have been concluded in spinal cord injury with MSCs. Mendonça et al. reported a remarkable improvement in tactile sensitivity and motor functioning upon intervention with MSCs, while Albu et al. found Wharton’s jelly MSCs to result in better pinprick sensation and improved bladder function. Other studies have also indicated enhanced sensitivity and motor power [319,320].

A hallmark of neurodegenerative illnesses is the gradual loss of neurons, which culminates in their death, with symptomatic treatments but no cure. This situation has driven investigators toward regenerative strategies, including the potential application of MSCs, which can be induced to acquire a neural phenotype in vitro. In terms of nerve repair in vivo, preclinical and clinical research are still needed to evaluate their effectiveness. MSC therapy has been demonstrated in certain clinical trials to increase survival rates, decrease pathology, maintain cognitive abilities, minimize relapse frequency, and relieve symptoms [321]. Among the disorders under trial are Alzheimer’s disease, amyotrophic lateral sclerosis, multiple system atrophy (MSA), Parkinson’s disease, and spinal cord injury.

Parkinson’s disease (PD) is one of the leading causes of morbidity and death, characterized by the loss of dopaminergic neurons [322]. MSC treatment has been demonstrated in preclinical animals to enhance motor function and reduce neuroinflammation concerning this problem. For instance, umbilical cord mesenchymal stem cells (UC-MSCs) and Wharton’s jelly mesenchymal stem cells (WJ-MSCs) improved locomotor deficits and restored brain-derived neurotrophic factors [323,324]. MSC-derived exosomes also reduced dopaminergic neuron loss and increased dopamine levels [325]. These research works pointed to the possibility of MSCs for the treatment of Parkinson’s disease. Additionally, promising results with MSC therapy have been shown. In a controlled, randomized clinical study, the intra-arterial infusion of BMSCs was well tolerated and reduced disease progression [326]. Stable motor function in PD patients was reported by Canesi et al., while Jaillard et al. found improved motor function and neuroplasticity in patients with stroke treated with BMSCs [327,328]. Overall, BMSCs offer considerable potential for neurological diseases, opening new possibilities for clinical treatment.

Alzheimer’s disease is a result of the destruction of certain cells in the brain leading to dementia and affects approximately 6.2 million people in the U.S. [324,329]. Investigations regarding the potential of MSCs for slowing down the progression of the disease and regenerating damaged neural tissue are currently being conducted. Studies have demonstrated that umbilical cord blood-delivered MSC (UCB-MSCs) reduces β-amyloid plaques and promotes neurogenesis [330]. Besides, the therapeutic potential of MSC-derived exosomes and menstrual blood-derived MSCs has been suggested to have potential medicinal uses in improving cognitive function and reducing amyloid plaques [331,332].

Multiple-system atrophy is a fatal neurodegenerative progressive disorder. Its prevalence is around 4.4 per 100,000 subjects [333]. Some promise in the treatment of MSA has been noted with MSCs, whereby intra-arterial BM-MSCs benefited patients at medium and high doses, with better outcomes and slower disease progression [334]. Long-term benefits in the amelioration of MSA symptoms have been realized in studies using UCB-MSCs. In the models of MSA, MSCs reduced polyamine-induced damage [335,336]. Clinical trials using autologous adipose-derived mesenchymal stem cells (Ad-MSCs) have also shown a reduction in the progression of the disease, without side effects [337]. However, high dosages are reported to have harmful effects on MSCs, and optimal dosing is not yet studied elaborately [338].

In ALS, when motor neurons are affected and progressive paralysis results, some trials with MSC treatments seem to show that they are safe and effective [339]. It has been reported that clinical trials of MSCs in ALS showed improvement in ALS Functional Rating Scale scores and survival times, with some showing increased anti-inflammatory cytokines and reduced disease progression when combined with Riluzole [340]. These observations thus support the potential use of MSCs in the treatment of ALS.

MSCs are under investigation for restoring function loss due to spinal cord injury. Clinical trials in humans have reported that intravenous UC-MSCs and MSCs associated with NeuroRegen scaffolds were able to promote motor and sensory functions [341,342]. Intrathecal Ad-MSCs induced improvement of motor skills and recovery. In animal models, MSCs labeled with nanoparticles demonstrated the production of nerve growth factor and recovery [343]. These results further reiterate the potential of MSC therapy in settings of spinal cord regeneration and neural repair. These data collectively demonstrate huge potential for MSCs in treating a variety of neurodegenerative disorders and offering new opportunities for clinical interventions.

### 7.4. Electrical and Magnetic Stimulation

Electrical signals in neuronal communication, survival, differentiation, and functional expression are considered essential. According to available information, it is established that electrical signals play vital roles in neurogenesis, tissue repair, and the general growth of neural tissues. Induced electric fields have been demonstrated particularly to influence neuronal activities, showing potential for disease mitigation and neural regeneration [344].

Two common forms of magnetic fields that have been utilized are the static magnetic field (SMF) and pulsed magnetic field (PMF), applied in studies about nerve cells. It has been reported that PMFs can successfully enhance nerve regeneration without any side effects [345,346]. SMF is a 0 Hz frequency with constant intensity and direction; recently, the study of the cellular effect of this was conducted, showing that the effects of SMF on cellular proliferation vary between different cell types. Most of these studies did not reveal any significant difference in cell cycle distribution between the SMF exposed and the control groups [347]. 

One of the mechanisms by which magnetic fields exert their action on the cells is related to the changed ion-specific channels in excitable membranes [348]. High-intensity magnetic fields change the orientation of the diamagnetic organic molecules, while low-intensity fields change the structure of the membrane. The voltage-gated ion channels are particularly sensitive to magnetic fields, especially the potassium, sodium, and calcium channels, making neurons very responsive to such fields [349]. However, studies have returned divergent effects on the flow of calcium ions, where Aldinucci showed that human lymphocytes subjected to SMFs and PMFs increased the influx of Ca^2+^ ions [350]. In addition to the role of calcium ion channels in synaptic functions due to magnetic fields, Ben Yakir-Blumkin et al. observed that SMFs inhibited apoptosis of cortical neurons by stimulating the influx of Ca^2+^ through L-type channels [351].

Studies have revealed that endogenous electrical currents in the order of the average field strength of approximately 3 V/m can guide neuroblast migration in the adult mouse brain [352]. This is similarly improved in neuronal mobilization and differentiation dependent on voltage and duration with exogenous electrical stimulation. New research has utilized implantable conductive platforms to investigate the different electrical effects on neuronal cells for the potential treatment of neural injuries. Implantable conductive platforms have been used to deliver electrical stimulation to transplanted neural progenitor cells in attempts to improve outcomes in both peripheral nerve injury and stroke models. Cell therapy with electrical modulation is bringing new, exciting avenues in the domains of neuronal regeneration and repair [344]. Peripheral nerve regeneration relies on neurite outgrowth and myelin sheath formation. One of the key mechanisms is the magnetic force acting on the target cells, in particular Schwann cells. Mann et al. reported that nuclear magnetic resonance therapy (NMRT) increases Schwann cell proliferation and enhances neuronal maturation and neurite outgrowth, supporting dorsal root ganglion (DRG) neuron survival [353]. SCs could self-produce their stimuli to secrete basal lamina, thus increasing the mechanical resistance [354]. The activation of mechanical stimulation initiates pathways and increases the expression of β1 integrin, promoting the proliferation of SCs [349]. Xia et al. isolated miR-23b-3p from SC-derived exosomes as a key element in mechanical stretch and nerve regeneration [355]. Other force-generating factors include neurotrophic factors, such as netrin-1 and NGF, which have roles in force generation for axonal regeneration [356].

Studies indicate that the cell growth direction in response to magnetic fields varies by cell type. For instance, Macias et al. demonstrated that DRG neurons grow parallel to the electric field, while Eguchi et al. found that SCs and collagen grow in different directions under strong SMFs (8-T), with SCs aligning parallel and collagen perpendicular to the field [357,358].

Other cells, such as fibrin, PC12 cells, erythrocytes, and osteoblasts, also align parallel to magnetic fields, which could be significant for guiding peripheral nerve regeneration [347]. However, strong magnetic fields can damage cells and cause genetic mutations [359]. Liu et al. found that high-intensity magnetic fields might hinder SC growth, with safe PMF frequencies at 0.5, 1.0, or 2.0 mT [360]. Eguchi observed normal SC and collagen growth under strong SMFs (8-T), suggesting that SCs may tolerate SMFs better than PMFs [358].

Additionally, magnetic hydrogels, as a form of an advantageous approach to neural regeneration, have been explored due to their ability to enhance cellular properties through magnetic stimulation. For example, NSCs on collagen hydrogels with magnetic iron oxide (Fe_3_O_4_) nanoparticles showed improved gene delivery and increased green fluorescent protein levels through magnetofection, indicating its potential for neural applications [361]. 

Research using superparamagnetic iron oxide nanoparticles (SPIONs) revealed that NSCs with high SPION concentrations formed more neuro-spheres in the absence of a static magnetic field but fewer neuro-spheres under a static field of 50 ± 10 mT [362].

Directed axonal development and cell differentiation are further enhanced by magnetic stimulation. Human neuroblastoma SH-SY5Y cells on collagen hydrogels containing magnetic graphene oxide and iron oxide nanoparticles showed improved neurite growth and orientation toward the magnetic field [363]. Similarly, collagen hydrogels with gold magnetic nanoparticles directed an increase in neurite axons [364]. 

In scaffold design, magnetic nanoparticles support cellular adhesion and regeneration. Magnetic alginate microparticles in hydrogel scaffolds helped guide cellular remodeling, and similar approaches improved nerve regeneration in rats [365,366]. Magnetic nanoparticles also enable targeted delivery systems, such as magnetic nanogels guiding exosome delivery and NGF to specific sites, enhancing neural regeneration [367]. 

Nanofibers orientated magnetically offer stable structures for neurite growth and alignment. Research on magneto-responsive fibers in hydrogels has shown increased neurite length and alignment [368]. Silk fibroin/gelatin hydrogels with magnetic patterns also improved cell adhesion and neurite elongation under electrical stimulation [369]. 

Combining magnetic stimulation with piezoelectric materials offers advanced control over cellular functions. Micromotors with magnetic and piezoelectric nanoparticles can steer cells and induce differentiation via ultrasound [370,371]. Additionally, hydrogels with piezoelectric and magnetic nanoparticles have been used to improve axonal regrowth in models of spinal cord injuries [372]. This combination provides precise, targeted approaches for promoting neural repair and regeneration [362,371,372]. 

Another mechanism of magnetic nanoparticles (MNPs) is the magnetocaloric effect, promoting nerve regeneration through localized heating. Appropriate temperatures positively influence neurite growth [373].

Magnetic fields impact on growth and inflammatory factors: Zhang et al. found that low-intensity PMFs enhanced myelin protein expression and TGF levels in the CNS, while Mert observed increased nerve function and reduced chemokine expression with low-frequency PMF treatment [374,375]. Magnetic fields also influence neurotrophic factor mRNA expression, supporting nerve regeneration [376]. Finally, during nerve regeneration, macrophages change to an anti-inflammatory phenotype that supports the development of cells and the release of growth factors and ions [347]. Expanding on this, Dai showed that MNPs and alternating magnetic fields (AMFs) induced this change in macrophage phenotype, increasing the production and expression of IL-10 [377].

A study on SMF-exposed rat hippocampal neurons explored potential mechanisms for the response to magnetic stimulation by evaluating intracellular signaling pathways that were altered [378]. Liu considered mechanisms of remyelination and suggested that Raf-MEK-ERK and Rac-MKK-JNK-c-Jun pathways are implicated [379].

Further well-designed research is required to gain full insight into the magnetic effects on peripheral nerves, including all phenomena observed.

## 8. Applications, Case Studies, and Real-World Examples

Recent studies and clinical experiments have been carried out using focused ultrasound to open the BBB for improving neural regeneration and the treatment of neurological diseases. They are mainly classified, according to the used intensity, into high-intensity focused ultrasound (HIFU) and low-intensity focused ultrasound (LIFUS). HIFU generates very high temperatures and usually leads to protein denaturation and tissue coagulation at the focal point. On the other hand, when used in combination with microbubbles, LIFUS induced temporary and reversible modulation of the blood–brain barrier [380]. LIFUS can foster neurogenesis via the activation of endogenous neural stem cells within the adult brain. The effects were evident one week after LIFUS and had already diminished at four weeks [381]. A pilot study of eight subjects affected by Alzheimer’s demonstrated that focused ultrasound may transiently and reversibly open the BBB. The small number of eight participants recruited at only one establishment as part of an ongoing multicenter trial is one of the main limitations of this study. Further studies in larger cohorts are needed to further elucidate the FUS imaging responses [382]. FUS improves the shortcomings of stem cell therapy, such as low efficiency and uncontrollable differentiation. Its mechanical, cavitation, and thermal effects, with the aid of US-responsive particles, can enhance stimulation and drug delivery. Besides, it offers noninvasive imaging for deep tissue. It enhances the proliferation, differentiation, migration, and in vivo detection of stem cells; hence, it opened the window for several clinical applications [383]. FUS with microbubble agents has become a noninvasive, targeted approach for the temporary opening of the BBB, allowing local drug and gene delivery. Beyond mere delivery of known therapies, FUS-mediated disruption of the BBB also shows potential for neuromodulation and other beneficial physiologic responses [11]. Although the exact mechanism underlying opening of the BBB using FUS is not fully elucidated, it is perceived that physical oscillations of microbubbles impact on the vascular endothelial cells and the adjacent tissues. A study indicated that intravenous infusion of microbubbles and subsequent application of FUS to a targeted brain region resulted in acoustic cavitation, thereby leading to repeated contraction and expansion of microbubbles within the treated area [384]. With its safety and repeatability, FUS, therefore, remains a potentially important future treatment modality in neurological disorders [385]. Future improvements might envision an increased translation and delivery efficacy of mRNA through optimized designs of lipids or hybrid nanoparticles. Organ-targeted delivery options and biodegradable lipids will be the next innovative steps in the domain of mRNA-based therapies, further extending uses and impacting better health outcomes [386]. 

Lipid nanoparticles (LNP) have shown quite significant therapeutic efficacy in the RNA therapies’ delivery of nucleic acids and mRNA COVID-19 vaccines. They include types such as liposomes and solid-lipid nanoparticles, with applications in various industries. Nowadays, the most used technique is high-pressure homogenization. At the same time, dispersing of lipids in aqueous media is unpredictable [387]. LNPs have enormous potential for the treatment of various diseases by enhancing in vivo pharmacokinetic parameters of the encapsulated compounds, their transport, and chemical stability, while minimizing toxicity. Various substances of natural origin, from polyphenols to vitamins, antioxidants, dietary supplements, and herbs, are related to impressive health benefits but suffer mostly from limited solubility, stability, poor absorption, and fast elimination. Having encapsulated natural molecules in LNPs, we can link these with nanotechnology and realize the full medical and health potential that lies within [388]. Of the various lipid-based nanoparticles, liposomes and solid-lipid NPs have much potential to cross the BBB by means of receptor-mediated transcytosis and surface modification for the targeted CNS drug delivery for optimum therapeutic benefit [214]. One can easily imagine how new tools, such as the “BBB on a chip”, might further improve our understanding of BBB function post-TBI through mimicking barrier physiology under a range of pathological conditions, including but not limited to neuroinflammation or hypoxia, while enhancing insights into drug delivery and barrier disruption [389]. Further development in neuroimaging and new intravenous tracers may allow for improved measures of the breakdown of the BBB beyond the limitations of gadolinium-based MRI tracers. It remains to be elucidated how studies investigating its chronic nature clarify its connections to late comorbidities, such as Alzheimer’s disease and post-traumatic epilepsy. Therefore, future research is needed to determine relevant biomarkers that will enable early, noninvasive diagnosis of BBB damage in TBI patients [390]. Novel applications of nanoparticle-mediated intranasal delivery have shown improved outcomes in the treatment of TBI. Such modulation at the level of the BBB has allowed researchers to significantly improve the delivery of therapeutic agents directly into the brain. It was observed that such a method not only enables targeted drug delivery but also ensures enhanced neural regeneration. In this respect, the approach will become one of the promising developments in neurotrauma [391]. 

In addition to being a very effective strategy for treating brain injuries and neurodegenerative illnesses, this type of BBB manipulation also enhances neuronal regeneration. Treatments for brain injuries and neurodegenerative disorders may benefit greatly from BBB modification, which promotes neuronal regeneration. Open-label studies and clinical trials of methods, such as focused ultrasound, nanoparticle delivery, and intranasal delivery of drugs, have shown encouraging results in this respect. Stimulation of neurogenesis in the adult brain could be a very effective method for the treatment of a variety of neurodegenerative diseases and disorders. An expanding body of research indicates that electrical and magnetic stimulation, physical activity, environmental enrichment, dietary modifications, and pharmaceutical treatments can all be used to regulate neurogenic niches in a relatively safe way to promote neurogenesis [392]. These strategies are clearly less effective than more invasive therapies, such as stem cell transplantation, which are still purely experimental and possibly very risky [393]. Although considerable success has been achieved in inducing neurogenesis in animal models of various neurodegenerative diseases, the mechanisms are still multifaceted. Currently, most of these studies focus on ameliorating symptoms in already existing models but should, in the near future, also investigate the enhancement of neurogenesis in healthy subjects before disease onset to maintain brain functionality and prevent cognitive decline. This will not be easy, as findings from rodent models are difficult to translate to humans because of variations in the structure and function of the brain [394]. Furthermore, specific and reliable biomarkers and standard methods of neurogenesis measurement in humans are lacking, and large-scale clinical trials are having to contend with a host of ethical and regulatory complications [395].

## 9. Future Perspectives

The combination of BBB modulation with neural regeneration therapies has huge potential in neurological disorder treatments. In ischemic stroke, there is a disturbance in the integrity of the BBB, leading to several noxious molecules that are able to infiltrate the brain and induce nerve cell damage. After stroke, recovery of the BBB was considered highly important for neurological recovery in the brain [396]. A number of recent studies have been performed to enhance the repair of the BBB, which includes mainly the increase in tight junction protein expression and decreasing permeability, coupled with recovery and proliferation of endothelial cells. However, investigations on the restoration of other BBB components, such as pericytes, astrocytes, and basement membranes, are still very few [397,398]. Recent developments in nanotherapeutics against NDDs show huge clinical promise. During the past five years, a range of multifunctional nanocarriers have been designed to control the immunological response and inflammation process [398,399]. Gold nanoparticles have shown good promise in the treatment of neurological diseases, such as Alzheimer’s disease, Parkinson’s disease, and stroke. Besides neuroprotection, they exert anti-inflammatory action involving different mechanisms. AuNPs have potential for therapies against AD and PD and may help in stroke recovery by mitigating damages. Their effectiveness is supported by experimental models, suggesting innovative therapeutic strategies in neurology [400]. RNAi has opened new avenues for preclinical treatments suppressing particular pathways; hence, it brings new opportunities to siRNA-based nanotherapeutics. Nanogels provide noninvasive delivery and multifunctional capabilities for NDD treatment [401]. CRISPR-associated protein 9 provides frequently interspaced clusters of short palindromic repeats, facilitating gene editing and accurate treatment for diseases by correcting the mutation or knocking out deleterious genes [402]. Techniques modulating the BBB could already be used to deliver CRISPR-Cas9 to the brain, allowing gene editing to correct the genetic mutations underlying neurological disorders directly within neural cells. Several different promising methods facilitating the crossing of the BBB by therapeutic agents, including the CRISPR-Cas9 systems, are under development. This involves receptor-mediated transcytosis, nanoparticle delivery systems, and adeno-associated virus vectors [403,404]. However, how to effectively deliver it into the diseased cells is a major challenge. Gene therapy for neural regeneration offers huge promise but is hindered by significant challenges, particularly those related to viral vectors, which, while effective, have notable drawbacks. They can trigger immune responses, which may reduce the therapy’s effectiveness or cause adverse reactions. Some patients may already have immunity to the viruses used, further complicating treatment. Additionally, the vectors’ limited ability to specifically target the right neurons can lead to off-target effects. There is also the risk of insertional mutagenesis, where integrating the gene into the patient’s DNA could inadvertently cause cancer. Moreover, the capacity of these vectors to carry large genes is limited, and some only provide short-term effects, which is inadequate for chronic conditions requiring long-term treatment. Manufacturing these vectors at scale is another hurdle, making the therapy expensive and less accessible. While gene therapy holds tremendous promise for neural regeneration, the challenges associated with current viral vectors and broader issues related to delivery, safety, efficacy, and accessibility must be addressed to realize its full potential in clinical settings. Targeted delivery may be a focus in the future via the use of nonviral vectors [405]. 

Very few treatments exist for ischemic stroke. NSC’s offer a glimmer of hope for the restoration of brain functioning via both cell replacement and by providing support for endogenous repair mechanisms. However, the complex environment of the brain and low NSC survival make them less effective for treatment purposes [406]. Neuromodulation-based stem cell therapy presents a new, potent therapeutic approach, although it harbors challenges, such as target region identification, optimization of stimulation parameters, and minimalization of side effects. The current therapeutic potential of stem-cell-based brain repair can be significantly enhanced in theory by the supplementation of neuromodulation techniques, possibly achieving mutual boosting of both brain repair and stem cell potential, while creating favorable conditions for mutualistic survival. This combined approach can further increase neurorehabilitation, showing great promise for clinical uses [267,407]. Gene editing by CRISPR/Cas9 and nanocarriers could target specific genes, such as APOE4 in Alzheimer’s disease, for possible direct treatment. This strategy will be helpful because natural exosomes, capable of crossing the BBB, are supposed to have biocompatibility and offer targeted delivery into the brain areas involved in disease. Exosomes obtained from sources such as MSCs represent a new approach of precision medicine against neurodegenerative diseases [231]. 

Recently, critical roles of insulin have also been shown for the brain [408]. Astrocytes, microglia, and neurons all have insulin receptors expressed on them. These insulin-related effects on the central nervous system include metabolic functions, such as glucose uptake, and nonmetabolic functions, which enhance neuronal survival and reduce inflammation [409,410]. Insulin has the potential to enhance the survival of neurons and promote recovery after trauma or in neurodegenerative diseases [411]. Its anti-inflammatory properties benefit neurotrauma and neurodegeneration. TBI frequently leads to hyperglycemia from an acute stress response due to increased catecholamines, cortisol, and IL-6, possibly due to hypothalamic involvement. There is a correlation of hyperglycemia with lactic acidosis and an increased mortality rate. Hence, glucose monitoring in neurocritical care of TBI patients should be an important aspect in order to improve clinical outcomes. As such, systemically administered or even intranasal exogenous insulin is becoming an avenue of investigation that is increasingly pursued in the management of TBI, SCI, AD, and PD [412]. Though this field is very new, the future potential for combining BBB modulation with neural regeneration therapies is auspicious. If this were to be further developed and continued, then it would open new treatment paradigms that could provide improved treatment outcomes for patients suffering from neurological disorders and injuries.

## 10. Conclusions

Nanomedicine is an increasingly interdisciplinary field, and further progress in healthcare requires collaboration between the public and the private sectors. The main lines of research are standardization of therapies, improvement of the biological efficacy and safety of nanocarriers of drugs, and raising awareness of the potential of nanomedicine. Nanomedicine has become a transformative approach to neuroprotection, neuroregeneration, and BBB modulation. Some therapeutic approaches mediated by nanotechnology that have been applied to some pathologies include immunotherapy, stem cell therapy, and gene therapy, with applications also for the treatment of brain disorders. Such an application epitomizes the role of biocompatibility with functional versatility for gelatin-based hydrogels in providing a scaffold that promotes neural regeneration and serves as a vehicle for therapeutic delivery. Inclusion of guanidine moieties improved neuron viability, further demonstrating a role for chemical modifications in enhancing neural outcomes.

The distinct qualities of NPs improve the drug-free therapies of photodynamic treatment and radiation, solving problems of resistance after treatment and reducing mortality risks. However, for biosafety, a thorough evaluation of biodistribution, pharmacokinetics, and toxicity is required before implementation in clinical applications. Effective coordination, awareness, and evaluation of safety will push the nanomedicine field forward to real applications. Although very promising, results still face several challenges, as follow: a deeper understanding of the long-term effects of nanomaterials in the brain, potential toxicity, and developing standardized protocols to allow for clinical applications. It is, therefore, important to come up with standardized guidelines for NPs in medicine for their safe and effective usage. This would require national and international bodies to develop clear criteria for applying nanomaterials. Such standards would be enormously useful to researchers and greatly facilitate the translation of nanomedicine into clinical practice.

While nanomedicine holds great promise for brain drug delivery, overcoming the challenges posed by the BBB, NP design, immunogenicity, and regulatory issues requires continued innovation and interdisciplinary collaboration. Future research should focus on developing more sophisticated, targeted, and biocompatible NPs, alongside better understanding of brain disease heterogeneity and the mechanisms of NP interaction with brain tissues. Subsequent investigations must concentrate on meeting these challenges, optimizing nanomaterial properties, and exploring new therapeutic avenues. Advances in nanomedicine thus lead to great hope for the future in neuroprotection and neuroregeneration. Addressing these challenges will be critical to unlocking the full potential of nanomedicine in treating central nervous system disorders. If today’s limitations are overcome, ways of using the unique properties of nanomaterials will yield the path to new therapeutic strategies that eventually lead to better quality of life for patients with neurological disorders.

## Data Availability

No new data were created or analyzed in this study. Data sharing is not applicable to this article.
